# A deep learning approach to real-time Markov modeling of ion channel gating

**DOI:** 10.1038/s42004-024-01369-y

**Published:** 2024-11-30

**Authors:** Efthymios Oikonomou, Yannick Juli, Rajkumar Reddy Kolan, Linda Kern, Thomas Gruber, Christian Alzheimer, Patrick Krauss, Andreas Maier, Tobias Huth

**Affiliations:** 1 Institut für Physiologie und Pathophysiologie, Friedrich-Alexander-Universität Erlangen-Nürnberg, Erlangen, Germany; 2 Erlangen National High Performance Computing Center, Friedrich-Alexander-Universität Erlangen-Nürnberg, Erlangen, Germany; 3 Pattern Recognition Lab, Friedrich-Alexander-Universität Erlangen-Nürnberg, Erlangen, Germany

**Keywords:** Ion channels, Molecular modelling, Computational chemistry

## Abstract

The patch-clamp technique allows us to eavesdrop the gating behavior of individual ion channels with unprecedented temporal resolution. The signals arise from conformational changes of the channel protein as it makes rapid transitions between conducting and non-conducting states. However, unambiguous analysis of single-channel datasets is challenging given the inadvertently low signal-to-noise ratio as well as signal distortions caused by low-pass filtering. Ion channel kinetics are typically described using hidden Markov models (HMM), which allow conclusions on the inner workings of the protein. In this study, we present a Deep Learning approach for extracting models from single-channel recordings. Two-dimensional dwell-time histograms are computed from the idealized time series and are subsequently analyzed by two neural networks, that have been trained on simulated datasets, to determine the topology and the transition rates of the HMM. We show that this method is robust regarding noise and gating events beyond the corner frequency of the low-pass filter. In addition, we propose a method to evaluate the goodness of a predicted model by re-simulating the prediction. Finally, we tested the algorithm with data recorded on a patch-clamp setup. In principle, it meets the requirements for model extraction during an ongoing recording session in real-time.

## Abstract (web summary)

The patch-clamp technique enables probing of the gating behavior of individual ion channel proteins, but unambiguous analysis of single-channel datasets is challenging. Here, the authors present a deep learning approach for extracting Markov models from single-channel recordings.

## Introduction

Since the groundbreaking work of Hodgkin and Huxley^1^, who established a stunningly prescient model on how voltage-gated membrane conductance for Na^+^ and K^+^ shapes the trajectory of an action potential, understanding the gating behavior of the predicted ion channels has become a major scientific endeavor arguably culminating in the advent of the patch-clamp technique^2^. In its single-channel configuration, this method enables direct recordings of discrete movements of channel protein moieties associated with rapid transitions between conducting (open) and non-conducting (closed) states, thereby giving unparalleled insights into gating mechanisms at high temporal resolution^3^. Typically, hidden Markov models (HMM)^4^ are used to describe the underlying kinetics of ion channel gating^5–8^, which can be employed to deduce structure-function relationships.

One approach, among many, to infer the underlying HMM from a single-channel patch-clamp recording is the two-dimensional dwell-time histogram (2D-histogram) analysis^9^. It builds on the idealization of single-channel time series, that is, estimating in which conducting state (open or closed) the ion channel is at any given time. From the idealized time series, the durations of neighboring open and closed intervals (dwell times) are paired as tuples and are accumulated in the 2D-histogram. It has been shown that 2D-histograms contain all the necessary information to infer the underlying HMM of the recorded ion channel^8^. A major issue with approaches that encompass the idealization of the single-channel recording is their general susceptibility to noise and artifacts introduced by the limited bandwidth of the recording setup. Despite a number of advancements that have been made to improve the quality of the recordings^10,11^, noise is still the major limitation for analysis. Therefore, a low-pass filter is usually employed, which has the drawback of further reducing the bandwidth. In the end, the interplay of noise and bandwidth compromises the idealization process and especially the detection of fast gating events. Considering these effects, the temporal resolution was partially extended^12^. As an alternative, the direct analysis of single-channel recordings without idealization has also been explored^13–16^.

A considerable improvement to 2D-histogram analysis came with the introduction of simulations of single-channel time series^17,18^, where errors made during idealization of the recorded time series occur similarly in the simulated one. Therefore, errors partially cancel out in an iterative process of comparing both histograms to deduce the underlying HMM. Thereby, the modeling process becomes very robust regarding noise and fast gating. An improved version^19^ was later used to model the interaction of chloramine-T with the neuronal ion channel Nav1.2a^20^. In our recent study, we have demonstrated the superior performance of Markov modeling 2D-histograms using simulations^21^. High-Performance Computing (HPC) enabled us to manage the tremendous computational requirements of this approach and make the fit very accurate. With HPC, it is now possible to explore the minimum signal-to-noise ratio (SNR), recording bandwidth, and number of recorded gating events required for successful modeling. Nevertheless, the modeling process is limited by the available HPC resources and requires a certain amount of hands-on time to configure the algorithm. In this study, we present the solution to the latter problems by developing deep neural networks (NNs) using time-series simulations. We provide data showing that the artificial intelligence (AI) approach can compete with the previous 2D-Fit. In principle, after training the networks, this approach will enable online Markov modeling of single-channel patch-clamp recordings.

## Methods

### Simulating 2D-histograms

The process of transitioning between open and closed states of ion channels is termed gating. It is assumed to be an ideal process with the channels exclusively being either in a conducting or a non-conducting state. Transitions between states are considered to be instant on the relevant time scale. Experimentally recorded time series of single ion channels are distorted by noise and the transitions are affected by the low-pass filter of the setup as well as by the recording bandwidth of the amplifier. For the simulation of time series, a HMM is designated by the user. It is defined by a topology, which encompasses a number of open and closed states with given connections, and rates governing the transition between the states in a stochastic process^14,21^. First, an ideal time series is created with the HMM, meaning that samples are assigned to either an open or closed state for each sampling interval. According to the given conductances of the open and closed states, the current amplitudes are assigned. Then, a step response emulating the effects of the low-pass filter is applied to each transition between the current levels. Finally, to obtain a signal with the desired SNR, noise with an appropriate amplitude is added to the time series. The SNR is defined as

1 $${{\rm{SNR}}}=\frac{I}{\sigma }$$

with *I* being the current amplitude (difference between the open and closed current level) and *σ* the standard deviation of the noise. In this study, we did not account for open channel noise^22^ and assume the same σ for both the open and closed states. Unless otherwise stated, an SNR = 5 was used throughout the manuscript. The application of the step response and noise generation are described below.

In order to compute the two-dimensional dwell-time histograms (2D-histograms) used for training the NNs, the simulated time series are idealized using the higher-order Hinkley detector (HOHD)^23,24^. The HOHD takes the current amplitudes of the open and closed levels, as well as the SNR, as input. It computes higher-order integrals to derive a score, which is compared to an SNR-dependent threshold for event detection. Note, that the initial ideal time series generated with the HMM might deviate significantly from the idealized time series after application of the HOHD due to effects imposed by noise and filtering. Dwell-times of neighboring open and closed events are combined in tuples and assembled in a logarithmically binned 2D-histogram having a resolution of 60 × 60 bins with 10 bins per decade and ranging from 10 µs to 10 s. Since the time series used in this study are sufficiently long (more than 1 million samples), we assume detailed balance (microscopic reversibility), enabling us to use both: open to closed and closed to open dwell-time pairs in our 2D-histograms^25–27^. The resulting datasets (Table 1) are stored as NumPy arrays^28^.

**Table 1:** Training datasets

No.	Topology	Time series length [samples]	Range of rates [s^−1^]	SNR	Train size	Validation size	Test size	Generation	Noise type	Step response
1	Linear Five-State	10 M	10^2^–10^5^	5	10 M	180 k	180 k	simulated	experimental (patch)	experimental
2	COCOC	10 M	10^2^–10^5^	5	980 k	10 k	10 k	simulated	experimental (patch)	experimental
3	CCCOO	10 M	10^2^–10^5^	5	980 k	10 k	10 k	simulated	experimental (patch)	experimental
4	CCCOO	100 M	10^2^–10^5^	5	980 k	10 k	10 k	simulated	experimental (patch)	experimental
5	COCOC	10 M	10^2^–10^5^	2	980 k	10 k	10 k	simulated	experimental (patch)	experimental
6	COCOC	10 M	10^2^–10^4^ 10^4^–10^6^	5	980 k	10 k	10 k	simulated	experimental (patch)	experimental
7	COCOC	1 M	10^2^–10^5^	4–6	980 k	10 k	10 k	simulated	experimental (bath)	experimental
8	COCOC	1 M	10^2^–10^5^	4–6	980 k	10 k	10 k	simulated	experimental (bath)	4-pole Bessel
9	COCOC	1 M	10^2^–10^5^	4–6	980 k	10 k	10 k	simulated	lp-filtered white	experimental
10	COCOC	1 M	10^2^–10^5^	4–6	980 k	10 k	10 k	simulated	lp-filtered white	4-pole Bessel
11	COCOC	1 M	10^2^–10^5^	8–10	980 k	10 k	10 k	simulated	experimental (bath)	experimental
12	COCOC	1 M	10^2^–10^5^	8–10	980 k	10 k	10 k	simulated	experimental (bath)	4-pole Bessel
13	COCOC	1 M	10^2^–10^5^	8–10	980 k	10 k	10 k	simulated	lp-filtered white	experimental
14	COCOC	1 M	10^2^–10^5^	8–10	980 k	10 k	10 k	simulated	lp-filtered white	4-pole Bessel
15	COCOC	1 M	10^2^–10^5^	6	—	—	100	patch-clamp setup	—	—
16	COCOC	1 M	10^2^–10^5^	8	—	—	100	patch-clamp setup	—	—

The table lists the datasets that were used for training and evaluating the neural networks (NNs) and the parameters used for simulating data: Topology of the Markov model, length of the time series, range of the rate constants k_ij_, signal-to-noise ratio (SNR), size of the training dataset, and size of the dataset used for validation and testing. Generation refers to the acquisition of time series, which is either simulated or generated on a patch-clamp setup using ideal time series as voltage commands. The last columns state which noise and step response were used for the time series simulation: experimental noise, low-pass filtered white noise, step response of a digital 4-pole Bessel filter, or experimental step response (see methods).

The datasets were simulated on the Erlangen National High Performance Computing Center (NHR@FAU) parallel cluster “Fritz“, with each computing node containing two Intel Xeon Platinum 8360Y “Ice Lake” processors (36 cores per chip) running at a base frequency of 2.4 GHz, 54 MB shared L3 cache per chip and 256 GB of DDR4 RAM. The time consumption for generating training datasets is stated in Table 2.

**Table 2:** Time benchmarks for simulation of training data, training, inference, and model estimation with the 2D-Fit

Task	Hardware	Time consumption
Simulation of training data 1 million 2D-histograms time series length: 10 million samples	HPC-cluster (Intel Xeon Platinum 8360Y)	100 node-hours (72 cores per node)
Training 1 million training samples (2D-histograms) global batch size: 1024	HPC-cluster (NVIDIA A100 GPU)	2 node-hours (8 GPUs per node)
2D-Fit^21^ time series length: 10 million samples ensemble size: 64 population size: 800	HPC-cluster (Intel Xeon Platinum 8360Y)	16–48 node hours (72 cores per node)
Inference Single 2D-histogram	Desktop Computer (12th Gen Intel® Core™ i5-12600 @ 3.30 GHz with 6 cores)	67 ms ± 16 ms
Inference Single 2D-histogram	GPU (NVIDIA RTX Titan)	120 ms ± 40 ms
Inference 10,000 2D-histograms	GPU (NVIDIA RTX Titan)	<11 s (<1.1 ms each)
Model prediction and complete analysis calculating ${\bar{V}}_{{{\rm{D}}}}({{\bf{G}}},{{{\bf{H}}}}_{1},\ldots ,{{{\bf{H}}}}_{100})$, ${\bar{V}}_{{{\rm{R}}}}({{{\bf{H}}}}_{1},\ldots ,{{{\bf{H}}}}_{100})$, and uncertainty quantification 100 2D-histograms time series length: 10 million samples	(Workstation dual processor) Intel Xeon E5-2697 v2 @ 2.70 GHz	~30 s

The inference benchmarks, were made with the NN that was used for estimating the rates of a COCOC topology (Fig. 5A, C, E). The 2D-histograms were taken from its corresponding test dataset (Table 1 dataset No. 2; test data split). For the single 2D-histograms, the average and standard deviation of 1000 inference runs are displayed.

### Application of the step response

In the simulation process, the rectangular gating events of the ideal time series are replaced with the step response function emulating the effect of the low-pass filter and recording bandwidth of the recording system. The 2D-Fit implements two options for the step response. The first is the digital step response that was generated using a 4-pole low-pass Bessel filter function with the corner frequency set to 10 kHz from the Python library SciPy^29^. The second is the experimental step response that was recorded on the patch-clamp setup, similar to ref. ^30^. We recorded 1000 step responses and computed their ensemble average. The step responses were recorded at 100 kHz, with the gain set to 100 mV/pA, and the low-pass filter corner frequency set to 10 kHz. The resulting step response is 45 samples long.

### Generation of noise

After applying the step response to the ideal time series, a noise time series is superimposed. As for the step response, the 2D-Fit has two options for noise simulation. The first uses white noise that is subsequently filtered with a 4-pole digital low-pass Bessel filter at 10 kHz using SciPy^29^ and finally scaled to the desired SNR. For the second option, in order to reduce the mismatch between the digitally simulated data and real experimental recordings, noise is generated from a given power spectrum, as previously described^31^. The authors proposed to add a random phase to each point of a power spectrum to compute a randomized noise series using the inverse Fourier transformation. We implemented this feature into the 2D-Fit, using the Cooley-Tukey algorithm of the fast Fourier transform (FFT) and inverse fast Fourier transform (IFFT), which is an efficient implementation of the Fourier and inverse Fourier transformation^32^. The C++ source code for the Cooley-Tukey algorithm was kindly provided by Anda Ouyang (https://github.com/AndaOuyang/FFT). In total, more than 10 h of noise was recorded for the computation of the spectra, using a patch-clamp setup with an Axopatch 200B amplifier (Molecular Devices). Two datasets were acquired: One using the patch resistance of an Axon cell model (10 GΩ, Molecular Devices) and another with the bath resistance (10 MΩ). Additionally, to acquire a smooth power spectrum, we split the noise recordings of each set into 63 segments, computed the power spectrum of each segment, and then formed the ensemble average. With the underlying power spectrum, we are able to simulate noise time series with a length of 10 M samples. Finally, we acquired a noise power spectrum from a patch-clamp recording of a real cell. The recording took place at room temperature using a patch-clamp setup consisting of a CV 203BU headstage, an Axopatch 200B amplifier, and an Axon Digidata 1550B digitizer (all instruments from Molecular Devices). The time series were recorded using pCLAMP v11.2 (Molecular Devices). Borosilicate glass pipettes with filament (Science Products) were pulled on a DMZ-Universal Puller (Zeitz-Instruments) with a tip resistance of 21 MΩ. The patch-clamp data was collected from a HEK 293 T cell using the voltage-clamp configuration with near-physiological sodium and potassium ion gradients. After establishing a Gigaseal, the patch was excised, and the pipette was moved just beneath the surface of the bath solution. The time series was recorded at −90 mV, close to the equilibrium potential of potassium, at a sampling frequency of 100 kHz with the output gain set to 100 mV/pA and the built-in low-pass filter set to 10 kHz.

### Deep NN architectures, training, and evaluation

The NNs were trained on the datasets listed in Table 1. These datasets contain a number of 2D-histograms generated from different HMMs with randomly assigned transition rates drawn from a logarithmic distribution. The datasets were split into training, validation, and test data. During training, the validation data was used for monitoring performance. The test data, which was not used during training, was exclusively used for generating the figure plots. It has to be mentioned that, as for any Deep Learning approach, the NNs can only make predictions about objects that lie within the parameter space spanned by the training dataset. Nevertheless, using our pipeline, it is possible to train NNs covering the parameters space that one defines.

**Fig. 1:**
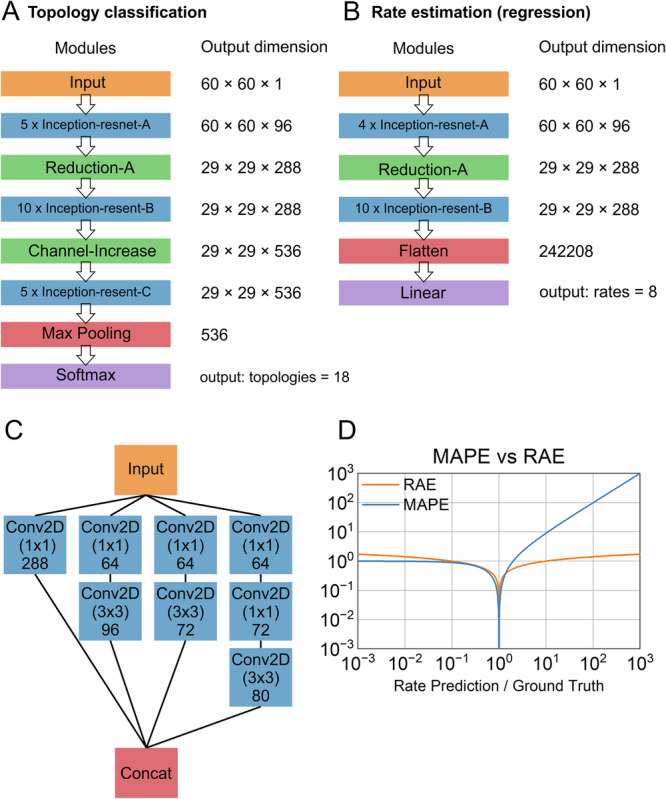
Illustration of the neural network architectures used for Markov modeling. In this study, we used modified versions of the Inception-Res-Net-V2 architecture^33^ for **A** topology discrimination and **B** rate estimation. **C** The original Reduction-B module was substituted with a module that increases the filter dimension without pooling. **D** Rate constant prediction was evaluated using the RAE error score (Eq. 3). In comparison to the mean absolute percentage error (MAPE), the RAE score is symmetrical with respect to the ground truth.

Two main architectures were implemented using TensorFlow 2.7.0 (DOI: 10.5281/zenodo.4724125) as illustrated in Fig. 1: One for the determination of the topology (Fig. 1A) and one for estimation of the rates (Fig. 1B). Both architectures are adapted versions of the Inception-Resnet-V2 as originally proposed by ref. ^33^. The architectures combine the technique of residual connections^34^ to allow for training of deep architectures, and inception architectures^35^. No batch normalization^36^ was used, since a drop in predictive performance was observed when enabled. Additionally, the number of filters in every convolutional layer was reduced by a factor of 4, the final Global-Average-Pooling-Layer was replaced with a Flatten-Layer and Global-Max-Pooling-Layer for the regression and classification tasks, respectively. All layers were initialized using a uniform Glorot initialization^37^ and all biases were initialized with zeros. We omitted the “stem” module, since it reduces the size of our 60 × 60 histograms too much, compared to the 299 × 299 sized images used in ref. ^33^. Furthermore, no dropout or any other forms of regularization were used. For the topology classification NN (Fig. 1A), the final layer was a dense layer with 18 output nodes, corresponding to the 18 linear five-state topologies to be classified, and a “softmax” activation. For the rates estimation architecture (Fig. 1B), the final layer was replaced with a dense layer consisting of 8 output nodes, which corresponds to the number of rates in the five-state topologies to be estimated, followed by a linear activation. Finally, the Reduction-B module was replaced with a module that increases channel size without pooling (Channel-Increase, Fig. 1C).

Training for all tasks was conducted using the Adam optimizer^38^ with a starting learning rate of (1e-3) for the first epoch and all other parameters as proposed previously^38^. For the regression and classification tasks, a global batch size of 1024 and 4096 were used, respectively. Unless mentioned otherwise, the learning rate was reduced by a factor of 0.1 after 8 epochs with no improvement in the validation loss, and an early stopping criterion was applied to terminate training after 12 epochs with no improvement in the validation loss. The selected loss functions were the categorical cross entropy and log-cosh for the classification and regression tasks, respectively.

As stated above, the bin width of the 2D-histograms was scaled logarithmically^39^. Furthermore, for training, bin occupancy was transformed according to:

2 $${a'}_{ij}=\left\{\begin{array}{cc}2{log }_{10}({a}_{ij}), & {a}_{ij} \, > \, 0\\ 0, \hfill & {a}_{ij}=0\end{array}\right.$$

with *a'*_*ij*_ and *a*_*ij*_ being the bin occupancy of the initial and rescaled 2D-histograms, respectively, of the bin with coordinates (*i*,*j*). This provides predictions of comparable performance to the canonical square root transformation^40^, while facilitating a faster convergence during training, likely due to compression of the occupancy of the 2D-histograms to a smaller range. Since the range of the rate constants *k*_*ij*_ that are to be estimated spans multiple decades, from 100 s^−1^ to 1 Ms^−1^, the labels for the regression task were also log-transformed for numerical stability of the training process.

The NNs were trained on the NHR@FAU parallel cluster “Alex“, with each node containing eight NVIDIA A100 (40 GB HBM2 @ 1555 GB/s; HGX board with NVLink). Multiple GPUs were used in parallel with distributed training and data parallelism, using the Tensorflow function tf.distribute.MirroredStrategy() with NcclAllReduce(). Time consumption for training the NNs and inference is stated in Table 2. The source code in its current stage (work in progress) is available at Zenodo (DOI: 10.5281/zenodo.12750594). Instructions on how to reproduce the data of this work and how to use the code for setting up experiments can be found in the Supplementary Methods.

### Rearrangement of label arrays to facilitate training for symmetric topologies

The label arrays store the ground truth for the rate estimation, which is used by the NNs during training. For symmetric topologies, for example, the linear COCOC topology (which can be read forward and backward), there are two ways to define a model given a label array. For example, if the eight transition rates are mapped to the indexes of the label array as [k_12_,k_21_,k_23_,k_32_,k_34_,k_43_,k_45_,k_54_], then they can be rearranged in reverse like [k_54_,k_45_,k_43_,k_34_,k_32_,k_23_,k_21_,k_12_] and still define the same HMM. Without a unique definition for each model, the networks have difficulties to train properly. Therefore, it was enforced that k_12_ > k_54_, and in the case of k_12_ < k_54_ the array was rearranged as stated above.

### Metrics for evaluating the performance of predicting the topology

The recall and precision are common metrics used to evaluate the performance of classification NNs. Let $D=\left\{{\mathrm{1,2}},...,18\right\}$ be the set of all indices of the topologies (classes). Then, ${r}_{i}=\frac{{n}_{{ii}}}{{K}_{i}}$ and ${p}_{i}=\frac{{n}_{{ii}}}{{L}_{i}}$ are the recall and precision of class with index $i\in D$, respectively, ${n}_{{ii}}$ the number of examples in the test dataset with ground truth $i$ classified as $i$ (correct classifications), ${K}_{i}$ the total number of examples of class $i$ in the test dataset, and ${L}_{i}$ the number of examples in the test dataset classified as $i$ by the NN. For evaluating the number of misclassifications, the analogous False Negative Rate (FNR) and False Discovery Rate (FDR) were used. They are defined as ${\text{FNR}}_{{ij}}=\frac{{n}_{{ij}}}{{K}_{i}}$ and ${\text{FDR}}_{{ij}}=\frac{{n}_{{ij}}}{{L}_{i}}$, respectively, with ${\text{FNR}}_{{ij}}$,${\text{FDR}}_{{ij}}$ being the FNR and FDR of the misclassification case that a class with index $i$ is classified as class $j\in {D\backslash }\{i\}$, and ${n}_{{ij}}$ being the number of examples in the test dataset with ground truth $i$ classified as $j$. The recall and FNR scores are related to the performance of the network from the developers’ perspective. The user (experimentalist) would rather be working with the precision and FDR scores since, in this case, they are equal to the posterior probability $P({y|x})$, with $x$ being the prediction of the NN and $y$ the ground truth, which indicates the probability of the class $y$ being the correct prediction given that the NN predicted class $x$.

### Metric for evaluating the performance of estimating the rates

In our previous work,^21^ the mean absolute percentage error (MAPE) score was used to assess the predictive performance of the algorithm related to the rate constants estimation. In this study, we use the root absolute error (RAE) score instead

3 $${{\rm{RAE}}}=\sqrt{\left|{\log }_{10}(k_{Pr})-{\log }_{10}(k_{GT})\right|}$$

with *k*_*Pr*_ being the prediction and *k*_*GT*_ the ground truth. A graphical comparison of the two scores is depicted in Fig. 1D.

### Computation of 2D-difference-histograms

When employing the proposed algorithm on real experimentally recorded data, the ground truth is unknown. Thus, the quality of the results cannot be evaluated directly. Nevertheless, an estimation of the predictions of the NNs without knowing the ground truth can be done using the difference histogram (2D_Diff_) that is computed with the experimental histogram (2D_GT_) and a histogram simulated using the prediction of the NNs (2D_Pr_). The 2D-histograms 2D_GT_ and 2D_Pr_ are scaled in the same way as the training data using Eq. 2, and then 2D_Diff_ is computed according to the formula:

4 $${z}_{{ij}}=\sqrt{{x}_{{ij}}^{2}-{y}_{{ij}}^{2}},\, {x}_{{ij}}^{2}-{y}_{{ij}}^{2}\ge 0$$

and

$${z}_{{ij}}=-\sqrt{{y}_{{ij}}^{2}-{x}_{{ij}}^{2}},\, {x}_{{ij}}^{2}-{y}_{{ij}}^{2}\, < \, 0$$

with ${z}_{{ij}},{y}_{{ij}},{x}_{{ij}}$ being the bin occupancies of 2D_Diff_, 2D_GT_, and 2D_Pr_, respectively, and $i,j$ the bin coordinates. We found that this representation allows for good visualization of differences in 2D_GT_ and 2D_Pr_. Practically, the errors of bins with small occupancies are suppressed, and those with a high occupancy are enhanced.

### Computation of the goodness of the predicted Markov model and uncertainty quantification of the corresponding transition rates

Based on Eq. 4, the normalized volume deviation ${V}_{D}$ between two 2D-histograms can be calculated

5 $${V}_{{{\rm{D}}}}({{\bf{S}}},{{\bf{M}}})=\frac{{\sum}_{i,j}\sqrt{\left|{s}_{{ij}}^{2}-{m}_{{ij}}^{2}\right|}}{{\sum}_{i,j}{s}_{{ij}}+{\sum}_{i,j}{m}_{{ij}}}$$

with ${V}_{{{\rm{D}}}}({{\bf{S}}},{{\bf{M}}})$ being the volume deviation between 2D-histograms ${{\bf{S}}}$ and ${{\bf{M}}}$, ${s}_{{ij}}$,${m}_{{ij}}$ the bin occupancy of ${{\bf{S}}}$ and ${{\bf{M}}}$, respectively, and $i,j$ the bin coordinates. Due to the normalization, ${V}_{{{\rm{D}}}}({{\bf{S}}},{{\bf{M}}})$ is constrained to the range [0,1], being ${V}_{{{\rm{D}}}}({{\bf{S}}},{{\bf{M}}})=0$ in case of a perfect match and ${V}_{{{\rm{D}}}}({{\bf{S}}},{{\bf{M}}})=1$ in case of no overlap.

However, each simulation as well as the experimental recordings encompass a certain variability due to their stochastic nature, the degree of which is dependent on the underlying HMM^41^. To quantify the goodness of a prediction and take into account the stochastic variability, $N$ time series are simulated using the predicted HMM and the 2D-histogram is calculated for each, obtaining $N$ 2D_Pr_-histograms (${{{\bf{H}}}}_{1},\ldots ,{{{\bf{H}}}}_{N}$). Then, the mean volume deviation ${\bar{V}}_{{{\rm{D}}}}({{\bf{G}}},{{{\bf{H}}}}_{1},\ldots ,{{{\bf{H}}}}_{N})$ between the 2D_GT_-histogram ${{\bf{G}}}$ and each of the $N$ 2D_Pr_-histograms ${{{\bf{H}}}}_{n}$, is calculated as

6 $${\bar{V}}_{{{\rm{D}}}}\left({{\bf{G}}},{{{\bf{H}}}}_{1},\ldots ,{{{\bf{H}}}}_{N}\right)=\frac{{\sum}_{n}{V}_{{{\rm{D}}}}\left({{\bf{G}}},{{{\bf{H}}}}_{n}\right)}{N}$$

with $n\in \left\{{{\mathrm{1,2}}},...,N\right\}$. Furthermore, to estimate the stochastic variability of the predicted model, the mean reference deviation ${\bar{V}}_{{{\rm{R}}}}({{{\bf{H}}}}_{1},\ldots ,{{{\bf{H}}}}_{N})$ between all $N$ 2D_Pr_-histograms ${{{\bf{H}}}}_{n}$ is defined as

7 $${\bar{V}}_{{{\rm{R}}}}({{{\bf{H}}}}_{1},\ldots ,{{{\bf{H}}}}_{N})=\frac{{\sum }_{n=1}^{N}{\sum }_{m=n+1}^{N}{V}_{{{\rm{D}}}}\left({{{\bf{H}}}}_{n},{{{\bf{H}}}}_{m}\right)}{N(N-1)/2}$$

with $n,m\in \left\{{{\mathrm{1,2}}},...,N\right\}$. The ${\bar{V}}_{{{\rm{R}}}}({{{\bf{H}}}}_{1},\ldots ,{{{\bf{H}}}}_{N})$ score can serve as a reference for ${\bar{V}}_{{{\rm{D}}}}({{\bf{G}}},{{{\bf{H}}}}_{1},\ldots ,{{{\bf{H}}}}_{N})$ to estimate the quality of a prediction.

Finally, to obtain an estimate for the uncertainty quantification of the transition rates of the predicted model, the $N$ simulated 2D_Pr_-histograms ${{{\bf{H}}}}_{n}$ are fed to the rates estimation NN to be re-predicted. This way, the parameter space around the initial prediction is explored, and a distribution, quantifying the uncertainty, for each transition rate can be obtained. Alternatively, scores based on the maximum likelihood score could also be defined^17,18,41^.

Note that all of the above calculations are independent of the ground truth model and can be obtained from the 2D-histogram of an experimental time series and the predicted HMM.

### Simulation of time series with the patch-clamp setup

One goal of this study was to evaluate the robustness of the NNs when dealing with experimental patch-clamp data. Time series were generated with the patch-clamp setup, as roughly outlined before^42^, to encompass the filter, bandwidth, and noise characteristics of “real” data. First, using a COCOC topology, ideal time series were simulated using the algorithm proposed before^14^. The ideal time series was then used as a command protocol for a voltage-clamp recording on an Axopatch 200B amplifier with a Digidata 1550B digitizer and Clampex 11.2 software (all from Molecular Devices). After compensation of capacitive artifacts, time series were recorded with a CV 203BU head stage connected to the bath resistance (10 MΩ) of a Patch-1U Model Cell (Molecular Devices). Higher resistances (Patch-configuration) could not be used for this purpose since significant capacitive artifacts would be introduced upon each voltage change in the command protocol. For analyzing time series of real cells, the cell-attached spectrum (Fig. 8B) should be used. Time series were recorded at a sampling frequency of 100 kHz, a gain of 100 mV/pA, and the low-pass filter of the amplifier set to 10 kHz. Capacitive feedback was enabled. Two sets of 100 time series each 10 s long were recorded and stored in a binary file format. Finally, 2D-histograms were computed using the HOHD^24^ as implemented in the 2D-Fit^21^ and saved as NumPy files. The simulated training datasets have a current amplitude of 2000 arbitrary units (AU) with the baseline set at 22,000 AU, the open level at 20,000 AU and are idealized using these values. On the other hand, the semi-synthetic datasets were recorded at ~33,000 AU and ~44,000 AU (with small variations depending on the given SNR) for the baseline and open level, respectively, which were used for the idealization.

### 2D-Fit

Simulation of time series and generation of 2D-histograms was performed using an improved version of the 2D-Fit program^21^. Modifications are related to noise generation and the step response function as stated above. For the employed HPC resources, see above and Table 2. The 2D-Fit algorithm was also used for evaluating the performance of the NNs. All settings were as described previously^21^. The source code in its current stage (work in progress) is available at Zenodo (DOI: 10.5281/zenodo.12750594). Instructions on how to reproduce the data of this work and use the code for setting up experiments can be found in the Supplementary Methods.

### Computation of inference benchmarks

The inference time was estimated in isolated environments created using Docker images provided by Intel (intel-optimized-tensorflow: 2.13-idp-base) and NVIDIA (nvcr.io/nvidia/tensorflow: 23.10-tf2-py3). These images are optimized for model inference on CPU and GPU, respectively. The results are summarized in Table 2. For inference of the single 2D-histograms the trained model was called directly with training set to False, while the 10,000 2D-histograms were fed to the trained NN in batches of 64 using the predict() function of the tensorflow.keras.Model class.

### Data analysis

The analysis and visualization of data were performed using Origin PRO2023 (OriginLab Corp).

## Results

Previously, we demonstrated the power of modeling single-channel patch-clamp recordings with two-dimensional dwell-time histograms (2D-histograms) using simulations (2D-Fit)^21^. 2D-histograms are an elegant way of squeezing rather large time series with varying lengths into a small and fixed-size data structure, which contains all necessary information to derive the underlying HMM^8^. For their computation, the time series are idealized using a jump detector, resulting in a train of consecutive “dwell-times” in either the open or closed state. The idealization has the additional advantage that noise, artifacts, and a drifting baseline can be handled during preprocessing. Then, neighboring open and closed dwell-times are paired and accumulated in the 2D-histogram. In our previous study, the underlying HMM was estimated in an iterative process. Single-channel time series were simulated using HMMs, and their transition rates were adjusted until the experimental and simulated 2D-histograms matched^21^. Different topologies had to be explored to find the overall best-corresponding model. The enormous computational effort was handled by utilizing high-performance computing (HPC).

**Fig. 2:**
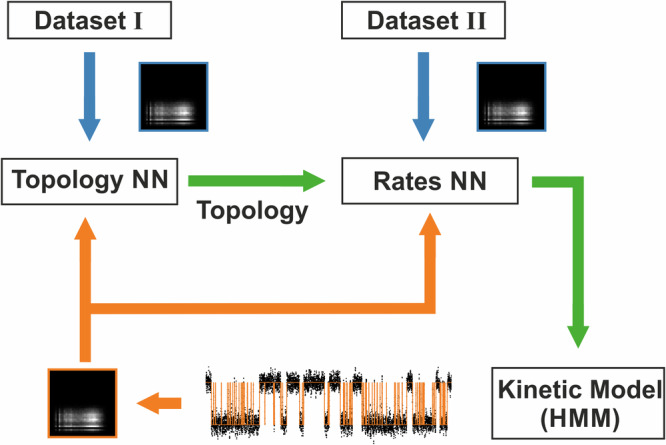
Flow chart of the proposed algorithm. The orange path shows the flow of the experimentally recorded data. It is sequentially fed to the topology estimation NN and to the NN for the estimation of the transition rates. The blue paths indicate training of the topology estimation NN and rates estimation NN with simulated training datasets I and II. Dataset I contains 2D-histograms simulated with a collection of models encompassing different topologies. Dataset II consists of a collection of simulated datasets were each set encompasses only one specific topology with a range of rates *k*_*ij*_. The green path shows the two stages of estimating the kinetic model. First, the topology is determined, and then the rates are estimated using the respective NN.

In this study, using the simulation routine of the 2D-Fit, sets of time series covering the desired parameter space were simulated, and 2D-histograms were computed to serve as training data (Fig. 2). Thereby, deducing the HMM from patch-clamp time series effectively becomes a task of image classification and analysis. Figure 2 illustrates the steps involved in extracting the kinetic scheme of a given experimental time series using NNs. After the idealization of the recorded time series, the resulting 2D-histogram (experimental 2D-histogram) is fed into a two-stage analysis. In the first stage, the topology of the HMM is estimated with the topology-NN. The second stage consists of a set of NNs each trained on a single topology from the first stage. The experimental 2D-histogram is fed to the specific NN of the second stage corresponding to the estimated topology in order to predict the rates. We used modified versions of the Inception-Res-Net-V2 architecture^33^ as illustrated in (Fig. 1A–C) for the NNs of both stages.

### NNs and simulation of training data

The training data for the NNs, a set of 2D-histograms, is derived from simulated time series. A smooth 2D-histogram that has a high bin occupancy with a low relative variation over neighboring bins is desirable for successful training. The stochastic variation per bin follows a Poisson distribution. Therefore, the relative errors ultimately depend on the number of gating events in the time series. For the experimental patch-clamp time series, the number of recorded events is limited by the gating behavior of the ion channel and the lifetime of the Gigaseal, ranging from minutes to tens of minutes. Additionally, the applied low-pass filter, in combination with the sampling rate, imposes a limit on obtained events per recording time. For the training data, the number of simulated events is limited by computational constraints such as available HPC resources. In our previous study, we analyzed the length of the time series for successful modeling with 2D-histograms^21^. Given typical gating behavior in the range of 10 s^−1^ to 100 ks^−1^, a length of at least 1 M samples (10 s at a sampling frequency of 100 kHz) was required to obtain meaningful results. Therefore, in this study, we decided to use time series consisting of 10 M samples (100 s simulated time), with the number of events in each varying strongly ranging from a few hundred up to more than 300,000, for training the NNs. The 2D-histograms computed from the data have a resolution of 60 × 60 with logarithmically scaled axes for both the open and closed dwell times, ranging from 10 µs to 10 s. The bin occupancy is scaled according to Eq. 2 to balance fast and slow rates generating a different amount of events in the time series. In our previous study, we used the mean absolute percentage error (MAPE) for evaluating fit results and comparing fit performance^21^. However, given a ratio of the predicted rate divided by the ground truth, the MAPE severely penalizes ratios above 1 and is not sensitive to small ratios below 10^−1^. In contrast, RAE behaves symmetrically regarding the ratio and does not lose sensitivity at low values. Therefore, in this study, we used the RAE (Eq. 3) instead, which we found is a better representation of the error (illustrated in Fig. 1D).

### Topology estimation of the underlying hidden Markov model

**Fig. 3:**
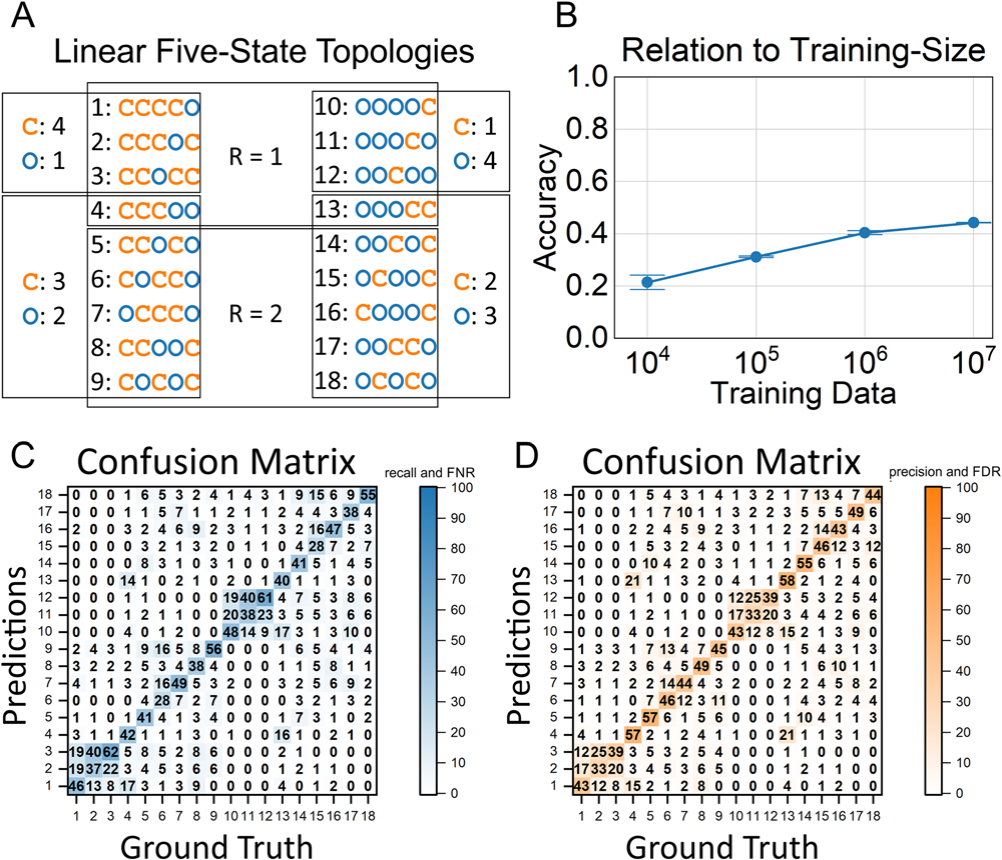
Topology estimation of Markov models using neural networks. **A** Shows all possible linear five-state topologies that were all encompassed in the training dataset for the topology estimation. They are grouped according to the number of open/closed states and their interconductance rank (number of independent C-O links). **B** The accuracy related to the size of the training dataset is displayed. The NN (Fig. 1A) was trained using subsets of dataset No. 1 (Table 1) with varying training dataset sizes. The training was repeated three times for each dataset, and the average, together with the standard deviation, is depicted. For the training dataset size of 10^7^, the patience for the learning rate reduction and training termination was reduced to 4 and 6, respectively. **C**, **D** The confusion matrices were obtained by testing a single NN that has been trained with 10^7^ 2D-histograms. The axes show the index of the topology, which can be gathered from (**A**). **C** The recall (diagonal values) and the False Negative Rate (FNR) (off-diagonal values) are displayed. **D** The precision (diagonal values) and the False Discovery Rate (FDR) (off-diagonal values) are displayed (see methods).

As detailed above, the first step in modeling the kinetics of ion channels is determining the topology of the underlying HMM. It has been shown that 2D-histograms contain all necessary information to infer the underlying HMM^8^. For the topology estimation, we simulated a training dataset encompassing all linear five-state topologies (Fig. 3A), comprising eight rates *k*_*ij*_ each. Figure 3A depicts the 18 topologies, grouped according to the number of open (O) and closed (C) states, respectively. Opposing topologies in both columns become identical when open and closed states are interchanged. Furthermore, the topologies are grouped according to their interconductance rank (R = 1, R = 2), which is defined as the number of independent C-O transitions in a topology^43^. For each topology, time series were simulated using transition rates drawn from a logarithmic distribution from within the range 100 s^−1^ to 100 ks^−1^. Using these time series, 2D-histograms were generated to train the modified Inception-Resnet-V2 architecture^33^ (Fig. 1A). First, we investigated how the accuracy varies in relation to the size of the training dataset. Training with each set was repeated three times. The obtained average accuracy is depicted in Fig. 3B. A considerable gain in accuracy with an increasing number of training samples was observed. For the largest dataset of 10^7^ 2D-histograms, an accuracy of ~44% was obtained (Fig. 3B), reflecting predominantly the inherent ambiguity of Markov modeling. Due to computational constraints, we did not simulate larger sets of training data. The resulting confusion matrices visualize the training results (Fig. 3C, D). The confusion matrix in Fig. 3C shows the recall (diagonal) and FNR, while Fig. 3D shows the precision (diagonal) and false discovery rate (FDR). The precision and FDR are especially useful for the experimentalist since, for any prediction of the NN, a probability distribution is obtained, constraining the set of likely topologies. Confusion, as indicated by the matrices, mainly occurred for certain topologies, within the same rank. As expected, opposing topologies from the left and right column (Fig. 3A) have near identical accuracy. In summary, the NNs were able to distinguish between different topologies. Nevertheless, there were substantial confusions between certain topologies, which we address in the discussion.

### Estimating the rates of the linear COCOC and CCCOO topologies

**Fig. 4:**
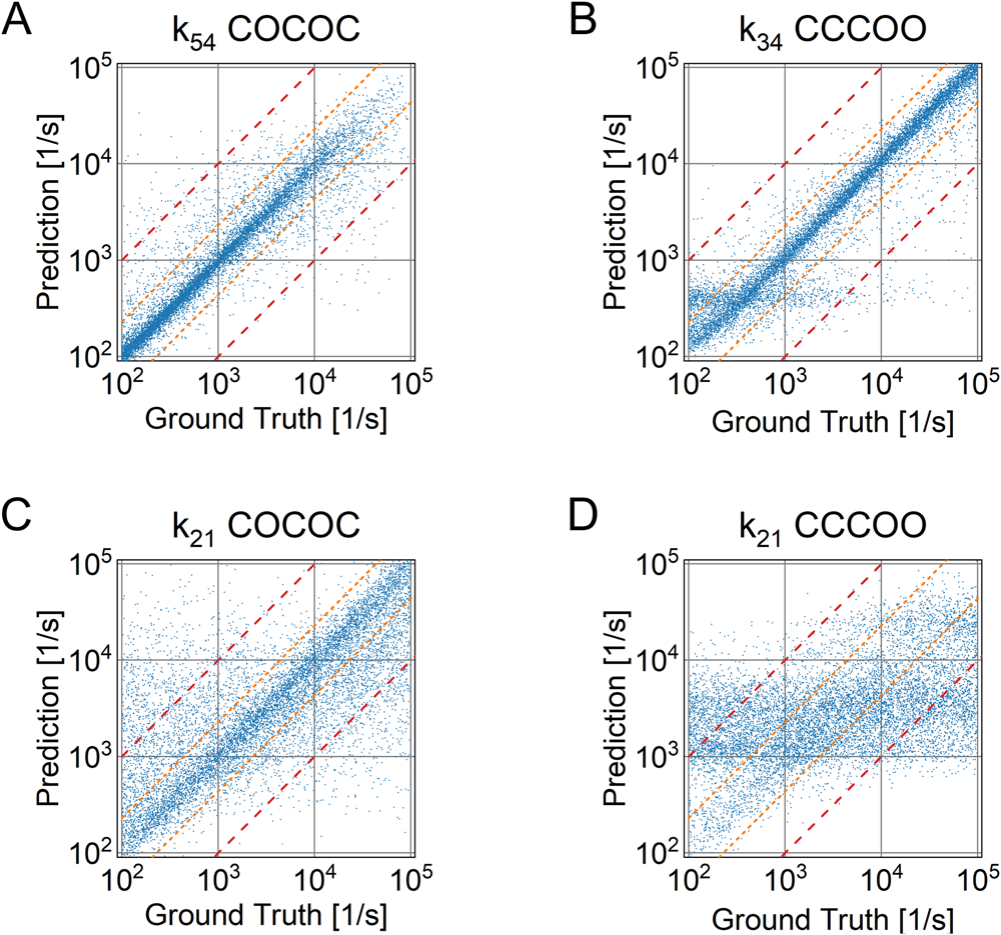
Transition rates estimation of COCOC and CCCOO models using neural networks. The regression architecture (Fig. 1A) was trained using datasets No. 2 and 3 (Table 1) containing models of the COCOC and CCCOO topologies, respectively. **A**–**D** After training, the network was evaluated using the test dataset. **A**, **B** illustrate the results of the predictions for the overall best-predicted rates *k*_*54*_, *k*_*34*_, and **C**, **D** for the worst-predicted rates *k*_*21*_, *k*_*21*_ according to the overall RAE score for topologies COCOC and CCCOO, respectively. Each test dataset contains 10,000 samples (2D-histograms). The orange short-dashed line and the red dashed line indicate the points on the graphs which have error scores (RAE) equal to 0.6 and 1.0, respectively.

After identifying the most likely topology of the underlying HMM of the time series, the rates that govern the transition between its states have to be estimated. For this task, individual NNs are used for each topology. Out of the 18 topologies (Fig. 3A), two were chosen to be analyzed in further detail by determining their underlying rates. The linear COCOC and CCCOO topologies represent two variants with rank 2 and rank 1, respectively. These topologies encompass eight rates *k*_*ij*_ each. The results of the best and worst predicted rates according to the error score (RAE, Eq. 3) are shown in Fig. 4A–D. For the COCOC topology, the best-predicted rate k_54_ shows a very good correlation with the ground truth (Fig. 4A). Due to the symmetry of the topology, the label array had to be rearranged by enforcing k_12_ > k_54_, as described in the methods section, to facilitate training. Therefore, values close to the maximum of the parameter space are less likely for k_54_. The rate k_21_ with the worst prediction still shows good correlation for this topology (Fig. 4C). The rates of the CCCOO topology should be more difficult to predict, since intraconductance transitions (C to C and O to O) do not produce observable events. Indeed, the result was less accurate compared to the COCOC models. Still, the C-O transition was predicted with a good correlation, albeit with several outliers at slower rates (Fig. 4B). However, the rate k_21_ connecting the distant C states is not predicted well with a considerable number of uncorrelated data points (Fig. 4D).

**Fig. 5:**
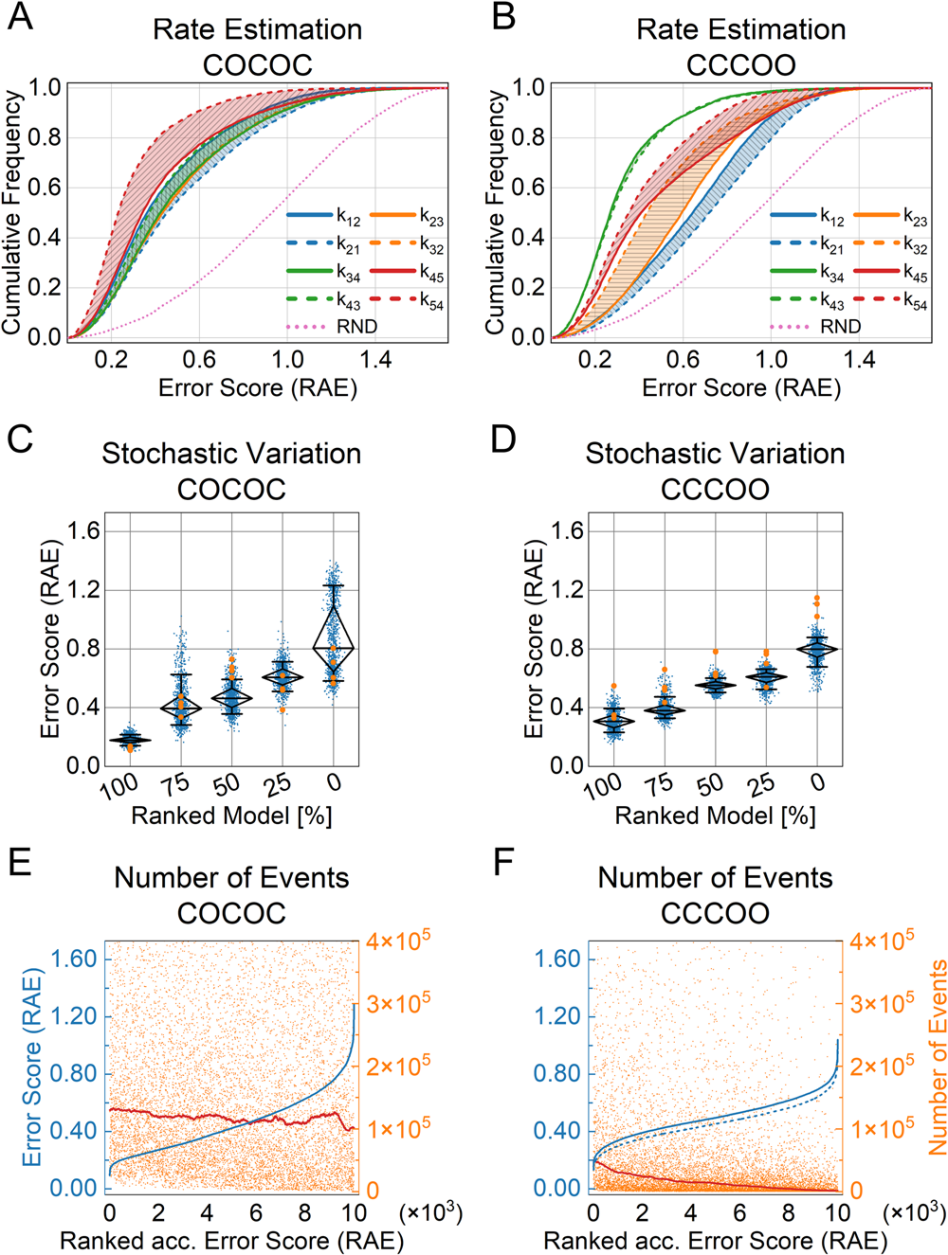
Analysis of the transition rates estimation for COCOC and CCCOO models. **A**, **B** Summarizes the prediction of all rates for the test datasets of the COCOC and CCCOO topologies (Table 1 dataset No. 2 and 3). The cumulative distributions of the error scores (RAE, Eq. 3) illustrate the predictive performance of individual rates. Rates connecting the same states are visually coupled together. For comparison, the dashed pink line shows the cumulative distribution error scores (RAE) of randomly predicted rates. The test dataset consists of 10,000 samples (2D-histograms), and the parameter space for the rates k_ij_ was 100 s^−1^ to 100 ks^−1^. **C**, **D** To investigate the impact of the stochastic simulation process, the predictions on the COCOC and CCCOO models were ranked according to the error score (RAE) and five models were selected from each topology at the percentile indicated on the horizontal axes. Each model was then simulated 1000 times using its ground truth and the rates were predicted with the respective regression NN. The averaged error scores (RAE) resulting from comparing ground truth and predictions of all eight rates from a given model are depicted. The diamond indicates the median as well as the 75 and 25 percentiles, while the whiskers denote the 10 and 90 percentiles. To compare the results with the 2D-Fit^21^, an additional four-time series were simulated for each model using the ground truths. For each time series, an ensemble of 64 runs was conducted with the 2D-Fit, and the RAE of the predictions with the highest likelihood for each time series are depicted as orange dots. **E**, **F** The ranked error scores (RAE) (blue line) are related to the number of detected events of their respective 2D-histogram (orange dots). In addition, the red line displays the moving geometric average over the number of events in the 2D-histograms, with a window size of 1025 samples. The dashed blue line in (**F**) indicates the ranked RAE error scores for the predictions of an NN that has been trained and tested on dataset No. 4 (Table 1), which contains 2D-histograms whose underlying time series had a length of 100 million samples.

To display the outcome of all predictions for the transition rates *k*_*ij*_ in a single graph for each topology, we introduced a different presentation for the results. Predicted rates *k*_*ij*_ are displayed as cumulative distributions of the error score (RAE). Rates connecting the same states have been paired and are visualized as the boundaries of the hatched areas. As a reference, the cumulative distribution of error scores that were computed with randomly drawn rates is displayed as pink dotted lines (Fig. 5A, B). With this representation, the disparity in predictive performance between the individual rates and the far better performance of the COCOC models in contrast to CCCOO becomes obvious (Fig. 5A, B). For the CCCOO topology the best-predicted rates were k_34_ and k_43_ (Fig. 5B), being the “gateway” states^43^ that facilitate the only C-O connection of the topology. Importantly, the distance of the error scores to the randomly drawn rates is evident nevertheless.

Simulations of single-channel time series, as well as gating of real ion channels are stochastic processes. Even with the same topology and identical rates, the resulting 2D-histograms vary slightly. Here we address the impact of this stochastic variation on the performance of the NNs. For both topologies (Fig. 5A, B) the predictions of the transition rates were ranked according to their respective error score (RAE). Five models were selected from the 100th, 75th, 50th, 25th, and 0 percentiles. Using the ground truth for these models, 1000 2D-histograms were simulated each and the rates were predicted with the NNs. The error scores (RAE) of the predictions are visualized (Fig. 5C, D blue dots). For both topologies, the stochastic simulation process considerably influences the outcome of the NNs' prediction. While the prediction for the model at the 100th percentile is very robust, the variability increased for the other models at lower percentiles. For comparison, we analyzed the same models using the previously developed 2D-histogram fit with simulations (2D-Fit)^21^. Because of the considerable computational resources required we were only able to analyze a limited set of four time series of each model (Fig. 5C, D orange dots). Interestingly, the performance of both algorithms varies for the different models.

We anticipate, as stated above, that the smoothness of the 2D-histogram, depending on the number of events, has a fundamental impact on the quality of the predictions. To address this, we explored the performance of the NNs related to the number of detected events in the 2D-histograms. The ranked error scores (RAE) of the rates’ predictions were plotted together with the number of detected events in the respective 2D-histograms (Fig. 5E, F). Whereas for the COCOC no relevant correlation can be observed (Fig. 5E), for the CCCOO an inverse correlation between the number of detected events and the RAE score exists (Fig. 5F). Since the CCCOO topology has only a single C-O transition, it generates on average fewer events than the COCOC topology (Fig. 5E vs. 5F). Nevertheless, given the substantial variance in the number of detected events it is obviously not the only factor determining predictive performance. In order to estimate how much the predictive performance improves with increasing number of events, we trained another NN of the same architecture using dataset No. 4 (Table 1), which contains 2D-histograms whose underlying time series have a length of 100 million samples (roughly 17 min of simulated recording time). The results of the predictions on the test dataset are illustrated in Fig. 5F as the dashed blue line. As expected the error scores (RAE) improved compared to the NN for 10 million samples.

Overall, it can be stated that the rates of models underlying a linear five-state topology can be estimated using the Deep Learning approach. Predictive performance varies strongly across topologies and between rates. In addition, the rates of transitions that are farther away from C-O links are predicted worse than those that are closer. Given the same model, the stochastic variability of the simulations lead to significant variations in predictive performance. Finally, predictive performance may increase on average with the number of detected events depending on the topology.

### High noise and fast gating

Given the microscopic currents of ion channels, which are in the magnitude of fA to pA, patch-clamp recordings are always endowed with a significant amount of noise. To improve the SNR, a low-pass filter has to be applied, causing a distortion of the signal by imposing an effective limitation of the bandwidth. Transitions at rates higher than the corner frequency of the filter (fast gating) are particularly affected. The distortion manifests as an apparent reduction of the current amplitude. Hence, idealization of the time series may become inaccurate. In our recent publication, we have demonstrated that rates could still be extracted on a noisy background and beyond the corner frequency of the low-pass filter^21^. The basic idea is that errors made in the idealization occur similarly in the experimental and simulated time series and cancel out to a certain degree. Capitalizing on the same principle, we now probed the performance of NNs when employed on data with high noise and fast gating. We separately examine the effect of a high-noise background and fast gating. In both cases, the analysis is structured in the same way and is divided into two parts. First, we illustrate how the quality of single predictions can be assessed, and then we address the overall performance of the NNs.

**Fig. 6:**
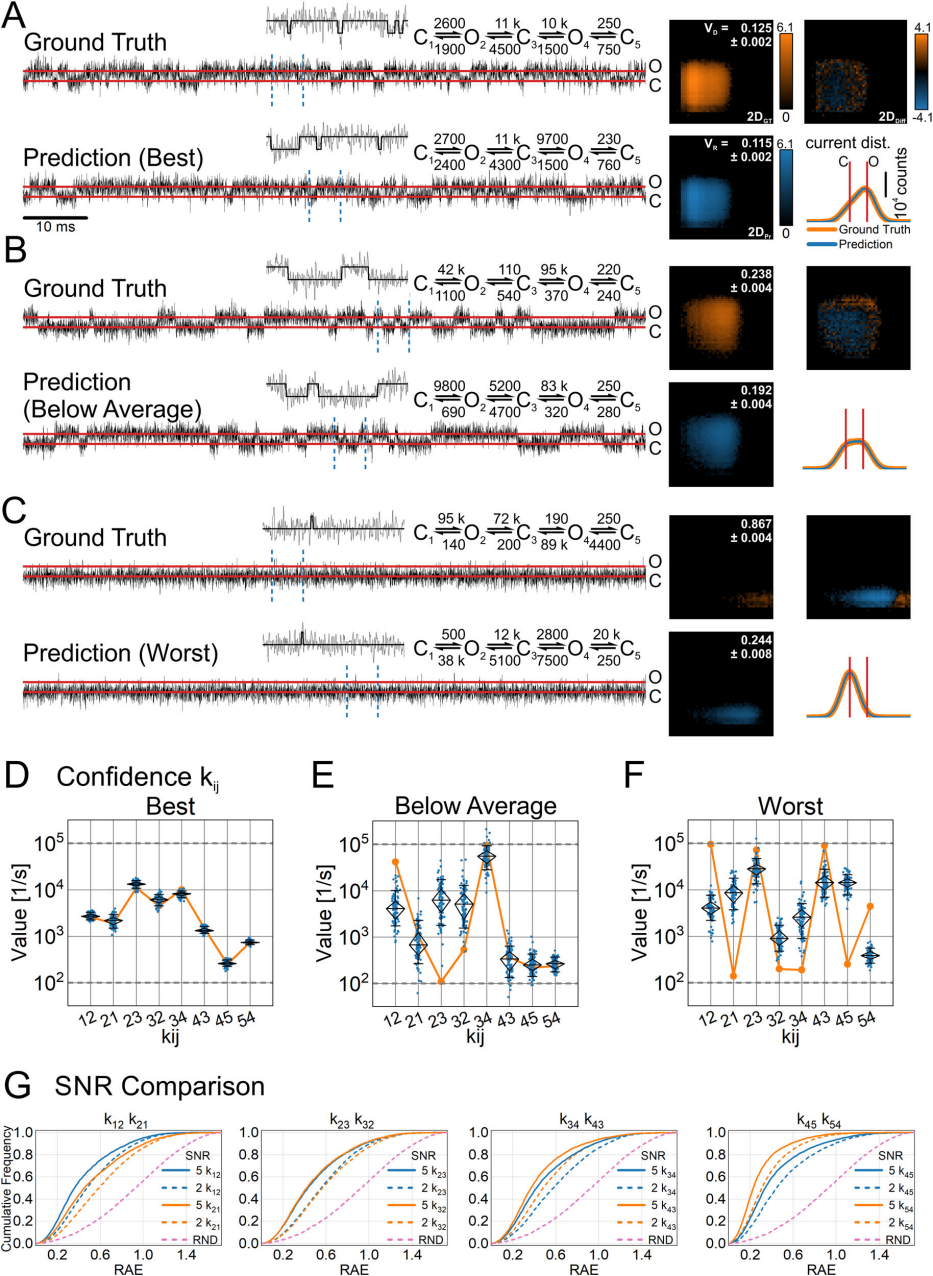
Evaluation of the predicted transition rates of time series with a low signal-to-noise ratio. Two datasets of 2D-histograms were generated with the COCOC topology (Table 1, datasets No. 2,5). The underlying time series had a signal-to-noise ratio of SNR = 5 and SNR = 2, respectively. The regression NNs were trained with the data, and the rates of the models in the test dataset were estimated. The predictions were ranked according to the averaged error scores (RAE) for each model. **A–C** Excerpts of time series simulated using the ground truth and predictions of the best-predicted model (**A**), a selected model approximately below the median (**B**), and the worst-predicted model (**C**) are shown. They are accompanied by their respective 2D-histograms (2D_GT_ and 2D_Pr_ for the ground truth and prediction). The closed dwell-times are represented on the horizontal axis and the open dwell-times on the vertical axis, ranging from 0.01 ms to 100 ms. For computing the 2D-histograms, time series with a length of 10 million samples were used. Furthermore, using time series with a length of 1 million samples, the current distributions of ground truth and prediction are plotted together in the same graph. The red lines indicate the open (O) and closed (C) current amplitudes, spanning 2000 arbitrary units (AU), with SNR = 2. In addition (inset), the segment of the time series between the vertical dashed blue lines is displayed with its corresponding idealization (black on gray). 100 simulations are computed with each predicted Markov model. The time series are idealized, and the 2D-histograms are generated. According to Eqs. 6, 7, the mean volume deviation and mean reference volume are then calculated (${\bar{V}}_{{{\rm{D}}}}({{\bf{G}}},{{{\bf{H}}}}_{1},\ldots ,{{{\bf{H}}}}_{100})$ and ${\bar{V}}_{{{\rm{R}}}}({{{\bf{H}}}}_{1},\ldots ,{{{\bf{H}}}}_{100})$, respectively). The volume differences are depicted in the 2D_GT_ and 2D_Pr_ histograms, respectively. Furthermore, distribution of the transition rates of the 100 re-predictions is displayed in (**D**–**F**). The horizontal dashed lines indicate the parameter range on which the NNs were trained. The diamond indicates the median as well as the 75 and 25 percentiles, while the whiskers denote the 10 and 90 percentiles. The orange dots connected with orange lines illustrate the ground truth as indicated in (**A**–**C**). **G** The predictions on all test datasets (10,000 for each SNR) are summarized as cumulative distributions of the error scores (RAE), for each k_ij_. Solid lines indicate the results for the data with an SNR = 5 and the dashed lines for an SNR = 2. For comparison, the dashed pink line shows the cumulative distribution of error scores (RAE) from randomly drawn rates within the parameter space of the datasets.

**Fig. 7:**
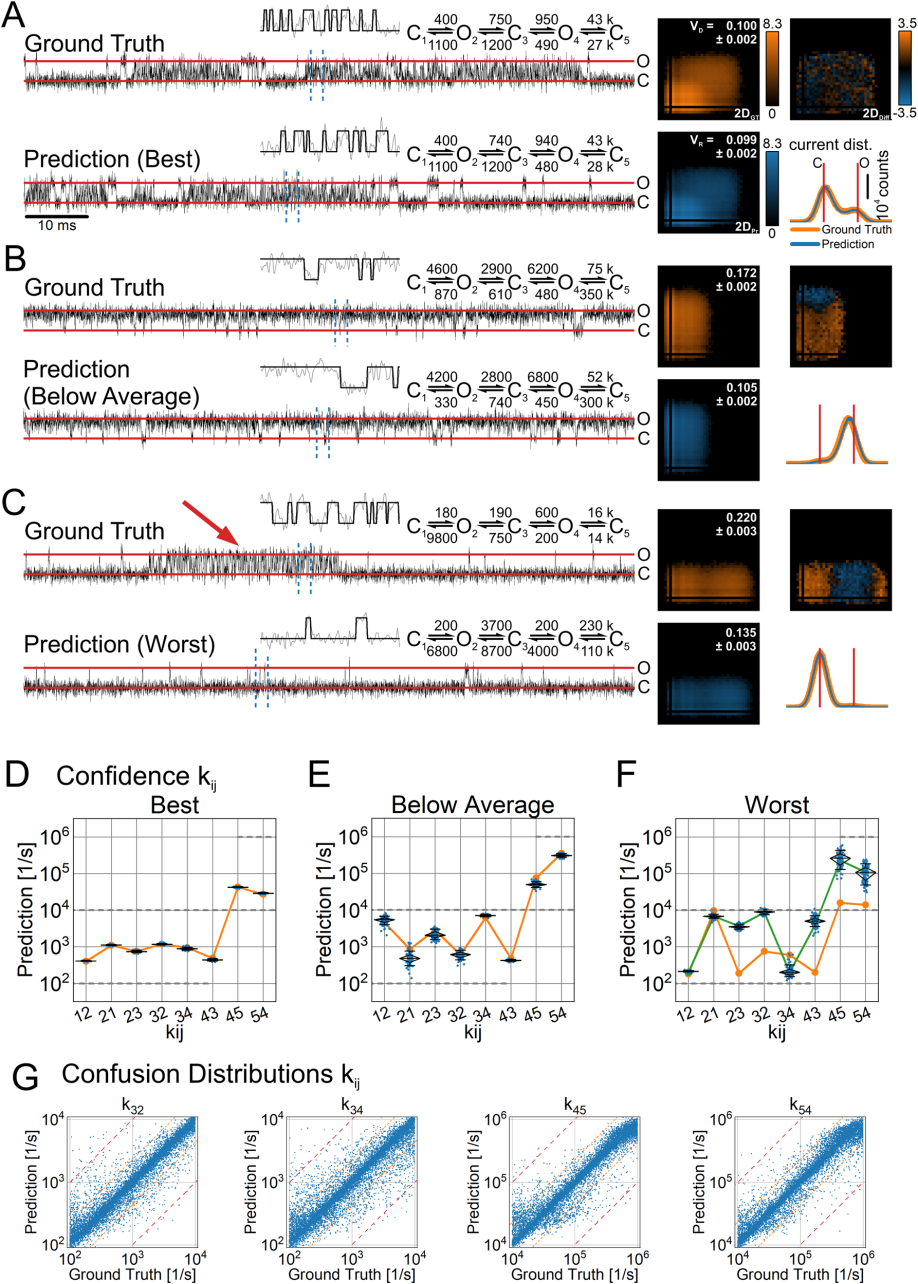
Transition rates estimation for COCOC models, including fast gating rates. Models were simulated with a COCOC topology, including fast rates that are considerably larger than the corner frequency of the low-pass filter (10 kHz). k_12_ to k_43_ were restricted to slower rates in the range of 0.1 ks^−1^ to 10 ks^−1^ and k_45_, k_54_ encompass the fast rates in the range of 10 ks^−1^ to 1 Ms^−1^. For this task, the regression architecture (Fig. 1B) was trained on dataset No. 6 (Table 1). The predictions were ranked according to the averaged error scores (RAE) for each model. **A**–**C** Excerpts of simulations using the ground truth and predictions of the best model (**A**), a selected model approximately below the median (**B**), and the worst model (**C**) are shown. They are accompanied by their respective 2D-histograms (2D_GT_ and 2D_Pr_ for the ground truth and prediction). The closed dwell-times are represented on the horizontal axis and the open dwell-times on the vertical axis, ranging from 0.01 ms to 100 ms. For computing the 2D-histograms, time series with a length of 10 million samples were used. Furthermore, using time series with a length of 1 million samples, the current distributions of ground truth and prediction are plotted together in the same graph. The red lines indicate the open (O) and closed (C) current amplitudes, spanning 2000 arbitrary units (AU), with an SNR = 5. In addition (inset), the segment of the time series between the vertical dashed blue lines is displayed with its corresponding idealization (black on gray). 100 simulations are computed with each predicted Markov model. The time series are idealized, and the 2D-histograms are generated. According to Eqs. 6, 7, the mean volume deviation and mean reference volume are then calculated, (${\bar{V}}_{{{\rm{D}}}}({{\bf{G}}},{{{\bf{H}}}}_{1},\ldots ,{{{\bf{H}}}}_{100})$ and ${\bar{V}}_{{{\rm{R}}}}({{{\bf{H}}}}_{1},\ldots ,{{{\bf{H}}}}_{100})$, respectively). The volume differences are depicted in the 2D_GT_ and 2D_PR_ histograms, respectively. Furthermore, distribution of the rates of the 100 re-predictions are displayed in (**D**–**F**). The horizontal dashed lines frame the parameter range on which the NNs were trained. The diamond indicates the median as well as the 75 and 25 percentiles, while the whiskers denote the 10 and 90 percentiles. The orange dots connected with orange lines illustrate the ground truth as indicated in (**A**–**C**). **F** The green dots connected with green lines illustrate the predicted values upon which the re-estimation is based on. **D**–**G** Scatter plots indicate the output of the NN on the test dataset. The orange dashed lines and the red dashed lines indicate error scores (RAE) equal to 0.6 and 1.0, respectively. **D**, **E** Show the results for the overall worst predicted slow rates (k_32_ and k_34_), while **F**, **G** show the results for the fast rates (k_45_ and k_54_).

For the SNR analysis, two NNs of the regression architecture (Fig. 1A) were trained with datasets No. 2 and No. 5 (Table 1) with an SNR = 5 (low noise) and an SNR = 2 (high noise), respectively. The models in the test dataset for the SNR = 2 were ranked according to the RAE of their predictions and the top-ranked, one selected from below the 50th percentile, and the lowest ranked were chosen. For each model, one time series was simulated using the respective ground truth labels. A small excerpt from each time series is depicted in Fig. 6A–C (Ground Truth). Similarly, three-time series were simulated with the predicted rates (Prediction). For each time series, the corresponding 2D-historgram (2D_GT_ and 2D_Pr_) is displayed accompanied by the 2D-difference-histogram (2D_Diff_) and the current distributions. For all three time series, the appearance of the predicted time series, the general shape of the 2D-histogram, as well as the current amplitude distributions are almost indistinguishable from the ground truth. However, the 2D_Diff_-histograms appear to be very sensitive to the quality of the prediction, mirroring the rank of the chosen model ranging from a perfect match (top-ranked, Fig. 6A), a fair match (Fig. 6B) to a considerable mismatch (lowest ranked, Fig. 6C). Of note, stochastic variations in the simulation of the ground truth, in the simulation of the predictions, and in prediction errors can contribute to an imperfect match. Furthermore, some models with different rates might exist that have very similar (almost indistinguishable) kinetics, leading to the NN predicting one of these alternative versions. This would result in a good match of the 2D_Pr_ and 2D_GT_ but substantial deviations from the ground truth. To address this issue, to quantify the goodness of the predicted model, and to obtain an uncertainty quantification of the predicted rates, we introduced the volume deviation score and the re-prediction of the rates (see methods). First, we re-simulated the predicted HMM 100 times to account for the randomness imposed by the stochastic simulation process and noise (SNR = 2). From the re-simulated time series, we yield 100 2D_Pr_-histograms $({{{\bf{H}}}}_{1},\ldots ,{{{\bf{H}}}}_{100})$ for comparison with the ground truth 2D_GT_-histogram, using the ${\bar{V}}_{{{\rm{D}}}}({{\bf{G}}},{{{\bf{H}}}}_{1},\ldots ,{{{\bf{H}}}}_{100})$ score (Eq. 6), which is displayed in the 2D_GT_-histograms (Figs. 6A–C and 7A–C). Theoretically, a value of ${\bar{V}}_{{{\rm{D}}}}({{\bf{G}}},{{{\bf{H}}}}_{1},\ldots ,{{{\bf{H}}}}_{100})=0$ would indicate an exact match and a value of ${\bar{V}}_{{{\rm{D}}}}({{\bf{G}}},{{{\bf{H}}}}_{1},\ldots ,{{{\bf{H}}}}_{100})=1$ non-overlapping histograms. However, due to the underlying stochastic processes, values close to zero cannot be achieved. This stochastic mismatch is model-specific^41^. Therefore, to estimate a reference by which to gauge the volume deviation, the volume reference deviation ${\bar{V}}_{{{\rm{R}}}}({{{\bf{H}}}}_{1},\ldots ,{{{\bf{H}}}}_{100})$ (Eq. 7) is computed using the re-simulated 2D_Pr_-histograms and is indicated in Figs. 6 and 7A–C in the 2D_Pr_-histograms. Additionally, by re-predicting solutions from the 100 re-simulated 2D_Pr_-histograms with the NNs, we explore the parameter space around the initial prediction and obtain the corresponding error scape of the rate constants (Fig. 6D–F). However, this only yields meaningful results if the mismatch between 2D_GT_ and 2D_Pr_ is sufficiently small, which is the case if ${\bar{V}}_{{{\rm{D}}}}({{\bf{G}}},{{{\bf{H}}}}_{1},\ldots ,{{{\bf{H}}}}_{100})$ is small and does not deviate strongly from ${\bar{V}}_{{{\rm{R}}}}({{{\bf{H}}}}_{1},\ldots ,{{{\bf{H}}}}_{100})$.

To exemplify how such an analysis could look like, we iterate through the presented model predictions. Given the 2D_Diff_ -histogram, the model in Fig. 6A represents an almost perfect match. Indeed, the deviation between the 2D_GT_-histogram and the 2D_Pr_-histograms is minimal (${\bar{V}}_{{{\rm{D}}}}({{\bf{G}}},{{{\bf{H}}}}_{1},\ldots ,{{{\bf{H}}}}_{100})=0.125$ vs. ${\bar{V}}_{{{\rm{R}}}}({{{\bf{H}}}}_{1},\ldots ,{{{\bf{H}}}}_{100})=0.115$), indicating that the prediction represents a very good solution. Next, the obtained distribution of rates *k*_*ij*_ from the re-predictions (Fig. 6D) can be inspected. The observed scatter of the *k*_*ij*_ is in the bounds of what can be expected from the variations caused by the stochastic nature of the simulation and the ion channel gating, as well as the low SNR = 2. Hence, this finding is compatible with the assumption that a unique model solution has been found. The model in Fig. 6B displays a slight degree of deviation between the 2D_GT_ and 2D_Pr_ histograms as indicated by the scores ${\bar{V}}_{{{\rm{D}}}}({{\bf{G}}},{{{\bf{H}}}}_{1},\ldots ,{{{\bf{H}}}}_{100})=0.238$ vs. ${\bar{V}}_{{{\rm{R}}}}({{{\bf{H}}}}_{1},\ldots ,{{{\bf{H}}}}_{100})=0.192$). Importantly, ${\bar{V}}_{{{\rm{R}}}}({{{\bf{H}}}}_{1},\ldots ,{{{\bf{H}}}}_{100})=0.192$ is considerably larger compared to the model in Fig. 6A, where ${\bar{V}}_{{{\rm{R}}}}({{{\bf{H}}}}_{1},\ldots ,{{{\bf{H}}}}_{100})=0.115$, suggesting that the ground truth model is stronger affected by the stochastic gating process and noise. The comparably large scatter of the re-predicted transition rates *k*_*ij*_ (Fig. 6E) supports this notion, and additionally indicates that the predicted set of *k*_*ij*_ is unlikely to be unique. Finally, the predicted model in Fig. 6C displays almost no overlap (${\bar{V}}_{{{\rm{D}}}}({{\bf{G}}},{{{\bf{H}}}}_{1},\ldots ,{{{\bf{H}}}}_{100})=0.867$ vs. ${\bar{V}}_{{{\rm{R}}}}({{{\bf{H}}}}_{1},\ldots ,{{{\bf{H}}}}_{100})=0.244$). Therefore, this model solution can be rejected.

Next, we analyze the performance of the NNs on the entire test set, using the RAE, which requires the ground truth. As in the preceding section, the results are displayed in the form of cumulative distributions (Fig. 6D–G). Each graph displays the results for both SNRs of a pair of rates that link the same states. Similarly to the previous section, the label array of the COCOC topology has been rearranged to facilitate the training of the NNs (see methods). As expected, the accuracy drops when reducing the SNR = 5 to SNR = 2, and the loss of accuracy is comparable for all k_ij_.

Now, the analysis of the results for fast gating follows. The regression architecture was trained using dataset No. 6 (Table 1), encompassing a five-state COCOC topology. Specifically, the slow rates k_12_ to k_43_ covered the range of 100 s^−1^ to 10 ks^−1^, while the fast rates k_45_ and k_54_ range from 10 ks^−1^ to 1 Ms^−1^, which solely contain frequencies that are above the corner frequency of the low-pass filter (10 kHz). For for illustrating the single predictions (Fig. 7A–C) we recapitulated the structure of presentation as described above (Fig. 6) for the SNR-analysis. A detailed description of how to estimate the quality of the predicted models is given above. The fast gating events are visible as episodes of flickering between the open and closed states or, in case of the model in Fig. 7B, as an apparent deviation from the open level. Qualitatively, we obtained similar results as for the noise analysis (Fig. 6A–C). Interestingly, for the lowest ranked model, rare episodes of fast gating (Fig. 7C, red arrow) were not captured by the networks, whereas “baseline gating” looks very similar.

In terms of network performance, the resulting prediction of the trained NNs on the entire test dataset is illustrated in Fig. 7D–G. The two slow rates (k_32_ and k_34_), having the worst predictions overall, are displayed and demonstrate a very good correlation with the ground truth (Fig. 7D, E). For the fast rates k_45_ and k_54_ the correlation is equally good (Fig. 7F, G). Importantly, even with a slightly worse accuracy beyond 300 ks^−1^ the information that those rates are very fast can still be retrieved. In conclusion, we demonstrated that NNs are capable of extracting rates on a noisy background (SNR = 2) and are not restricted to rates below the corner frequency of the low-pass filter. To demonstrate the capabilities of the algorithm, a comparison with an analytical approach is given in the Supplementary Results (Supplementary Fig. 1).

### Performance of the NNs on data obtained with a patch-clamp setup

**Fig. 8:**
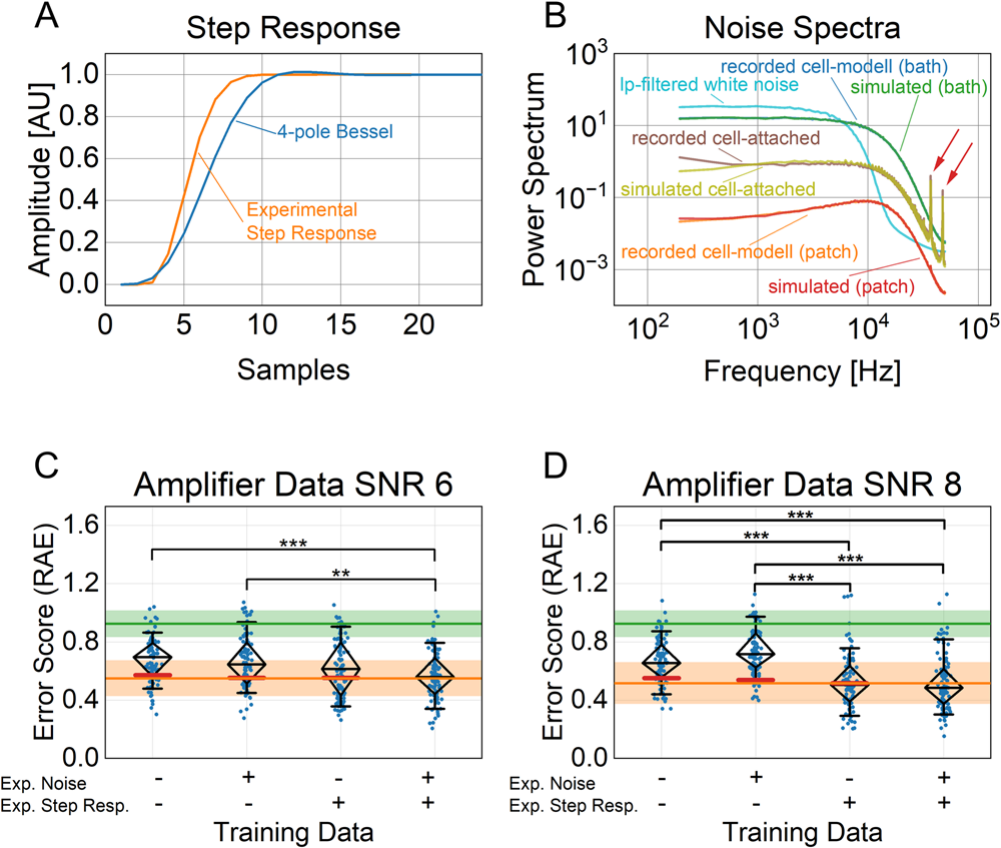
Transition rates estimation for data generated with the patch-clamp setup. **A** The experimentally derived step response of the patch-clamp setup considerably deviates from the simulated step response of a 4-pole low-pass Bessel filter. **B** Comparison of the power spectra of simulated and recorded noise. Noise has been recorded with different resistors of a cell model (blue: bath resistor, orange: patch resistor) and a real cell in the cell-attached configuration (brown), with the red arrows indicating stray noise. The simulated noise was generated with either white noise filtered with a digital 4-pole low-pass Bessel filter (cyan) or with the power spectrum of the corresponding recorded noise using the ^31^ algorithm (see methods, green: bath resistor, red: patch resistor, lime: cell-attached). **C**, **D** In total, eight NNs have been trained, each with a dataset containing a combination of a step response and noise type as indicated below the graph (Table 1 datasets No. 7–10 and 11–14, COCOC topology, with SNR = 4 to SNR = 6 and SNR = 8 to SNR = 10, for (**C**, **D**), respectively). Two sets of 100 time series with an SNR ≈ 6 and SNR ≈ 8 were recorded on the patch-clamp setup using ideal time series as voltage command protocols and the 2D-histograms were analysed with the respectively trained NNs. The diamond marks the median as well as the 25 and 75 percentiles, while the whiskers denote the 10 and 90 percentiles. Significance was tested with the Kruskal–Wallis-Test and pairwise with Dunn’s Test (**p < 0.01 and ***p < 0.001). The orange stripes indicate the predictions of the NNs on the test data that were not recorded on the patch-clamp amplifier, but was instead generated in the same way as the training data (Table 1 dataset No. 7 and 11 for (**C**, **D**), respectively). Note, the performance of the other NNs on their respective test datasets (Table 1 datasets No. 8, 9, 10 and No. 12, 13, 14 for (**A**) and (**B**), respectively) were very similar (red lines). The green stripes indicate the error scores for randomly drawn rates. The lines denote the medians and the boundaries of the stripes the 25 and 75 percentiles.

NNs can learn minuscule nuances that exist in training data, which could be important for high accuracy but could also be a confounding factor. Therefore, realistic simulations of patch-clamp data for training are critical to the performance of the NNs. By applying the 2D-histogram transformation, the dimensionality of the patch-clamp time series is reduced by rearranging a list of 1D-dwell times into a 2D array and removing the correlation of adjacent pairs. Therefore, the representation becomes more abstract and could reduce possible confounding details. Nevertheless, to minimize the mismatch between the simulated time series used for training and the experimental time series obtained via the patch-clamp setup, two significant improvements were made. First, the step response of the simulated data was optimized by recording multiple steps with the patch-clamp amplifier and computing their ensemble average, similarly to^30^. When comparing the recorded step response with the default simulated 4-pole low-pass Bessel filter, deviations between the two are readily visible (Fig. 8A). The recorded step response has a steeper slope and fewer oscillations. We replaced the default Bessel filter response with the experimental one and used it in the simulation process. The second improvement was to match the noise spectra of the experimental time series with the simulated one. The noise was recorded with the amplifier of the patch-clamp setup, and the power spectrum was computed. Simulated noise was then generated from the power spectrum using an algorithm proposed previously^31^. We recorded the noise from three different sources: a 10 MΩ resistor (bath) a 10 GΩ resistor (patch) from a model cell, and a real cell in the cell-attached configuration (Fig. 8B). The power spectrum of all sources is distinctly different from the default artificial Gaussian white noise filtered with a 4-pole low-pass Bessel filter used in our previous study^21^. Using the power spectrum of the recorded time series, we were able to simulate noise with an indistinguishable power spectrum, including stray noise (red arrows, Fig. 8B), in the case of the real cell.

In this section, the robustness of NNs when applied to experimentally recorded single-channel patch-clamp data is analyzed. Since the ground truth of real recordings is unknown, semi-synthetic test datasets were generated. One feasible approach for accomplishing that is presented in ref. ^42^, where the patch-clamp setup is used to emulate real single-channel recordings. Using this method, we recorded single-channel data by executing waveform protocols with ideal time series on the patch-clamp setup. With this approach, we encompassed the noise spectrum and the filter response of the setup in the time series. However, in contrast to a real experimental time series, we know the ground truth. In total, two datasets consisting of 100 time series each with a length of 1 million samples (10 s) were acquired. The amplitude of the ideal time series was adjusted to obtain an SNR ≈ 6 and SNR ≈ 8, respectively (Table 1 datasets No. 15,16). After idealization, the resulting 2D-histograms were fed into NNs. The NNs were trained on fully synthetically simulated datasets generated using different combinations of the default step response, default noise, experimental step response, and experimental noise. For the time series with an SNR ≈ 6, the NN trained on data between an SNR = 4 and SNR = 6 was generally able to generate meaningful results (Fig. 8C). Using the recorded step response and noise generated with the power spectrum combined to simulate the training data, did provide a significantly better result compared to the default, even reaching the performance of the respective simulated test dataset (orange colored line).

With an SNR = 8, the idealization of the time series for generating 2D-histograms is only negligibly affected by noise. Therefore, as expected, the type of noise did not affect the predictive performance of the NNs (Fig. 8D, trained with data between an SNR = 8 and SNR = 10). In contrast, using the experimental step response made a considerable difference.

In conclusion, it was demonstrated that the time series obtained with the patch-clamp setup can be successfully modeled. After accounting for the step response and the specific noise spectrum, the predictions did considerably improve to a level matching purely simulated data.

## Discussion

In this study, we demonstrated that NNs are capable of identifying the HMM that governs the gating kinetics of an ion channel. By capitalizing on the recent advancements made in massively simulating single-channel patch-clamp time series^21^, datasets consisting of two-dimensional dwell-time histograms (2D-histograms) were simulated and used for training NNs. With this Deep Learning approach, it is possible to identify the most likely topologies and estimate transition rates in a high-noise scenario, down to SNR = 2, as well as beyond the corner frequency of the low-pass filter. In principle, the trained NNs could be employed during an ongoing single-channel patch-clamp recording to obtain the kinetic model in real-time (see Table 2 for inference time).

The state of the art for topology identification is fitting the data to multiple topologies and then selecting the one that delivers the best results according to the fit score (e.g., log-likelihood)^44,45^. For analytical algorithms^12,13,46–48^, the computational burden this encompasses does not pose a problem, especially considering the computational power available today and given that they can be computed efficiently. However, when examining recordings with low SNR, which is related to a small ion channel conductance and fast gating behavior, the limits of analytical algorithms are approached^49^. The impact of low SNR, low-pass filtering, and consequently reduced recording bandwidth are not easily analytically resolved. That is where iterative simulation-based approaches excel^17–21^, since errors made in the idealization process, such as missed events and false alarms, cancel out by comparing simulations and experimental data. Unfortunately, this powerful method comes with a major drawback: substantial computational requirements. With the Deep Learning approach, we overcame the last hurdle. The simulations used to train the NNs need to be computed only once. Thereafter, the inference time for predicting the models is negligible (Table 2).

In our tests, the topology estimation NN reached an accuracy of ~44%. At first glance, this might seem unimpressive. However, a closer look at the confusion matrix (Fig. 3C, D) reveals that statistics limit confusion to only a few topologies. More specifically, the confusion matrix in Fig. 3D illustrates the precision and FDR scores, which equal the probability that any of the topologies is the correct one given a certain prediction of the NN. A group of the most probable topologies can be selected for further analysis and the transition rates can be estimated for each of them with the corresponding transition rates estimation NNs.

A common issue of data-driven approaches is evaluating the quality of an obtained result when the ground truth is unknown, which in our case consists of the topology and the rates of the HMM. Luckily, for single-channel recordings, this can be done rather easily by simulating the predicted model and comparing it to the experimental data. In Figs. 6A–C and 7A–C, we exemplify how such a comparison could look like. By visual inspection of the kinetics, the experimenter can already reject a result if there are obvious discrepancies between the two time series. Next, the current distributions of the time series can be overlaid, indicating if a match in the state occupancies has been achieved. This is especially helpful when examining ion channels with fast gating since the resulting skew in the current distributions contains increasingly more information as the transition rates rise beyond the corner frequency of the low-pass filter^50^. If this approach is extended to encompass non-stationary data, the ensemble of the re-simulated single-channel currents should match the time-dependent state occupancy ^20^. Finally, the most powerful tool for judging the goodness of an estimated solution is the 2D-histogram, since it comprises a complete visual representation of the unknown HMM^8^. Consequently, the 2D-difference histograms (2D_Diff_) proved to be very sensitive to prediction errors (Figs. 6A–C and 7A–C).

When evaluating the quality of a prediction, the concept of equivalent topologies has to be considered^43,51–53^. Models of different topologies exist that produce the exact same kinetic and are, therefore, indistinguishable. To this date, there is no analytical method to determine all topologies in a class of equivalent topologies^43^. By applying an analysis such as the one presented here, it is possible to end up with a solution that approximates the experimental data very well, meaning it has the same kinetic, but does not match the ground truth. If the deviation of the ground truth and predicted 2D-histograms (2D_GT_ and 2D_Pr_) are equally small for different topologies, the models could be structurally or practically equivalent.

As a quantifiable score of the goodness of an estimated model, i.e., the match of 2D_GT_ and 2D_Pr_, we introduce the mean volume deviation ${\bar{V}}_{{{\rm{D}}}}({{\bf{G}}},{{{\bf{H}}}}_{1},\ldots ,{{{\bf{H}}}}_{100})$ (Eq. 6) and mean volume reference ${\bar{V}}_{{{\rm{R}}}}({{{\bf{H}}}}_{1},\ldots ,{{{\bf{H}}}}_{100})$ (Eq. 7) (Figs. 6A–C and 7A–C). The scores are calculated by simulating a set of 100 time series using the predicted model and computing the 2D-histogram for each, incorporating the stochastic variability of the simulation process. A complete match ${\bar{V}}_{{{\rm{D}}}}({{\bf{G}}},{{{\bf{H}}}}_{1},\ldots ,{{{\bf{H}}}}_{100})=0$ cannot be achieved given the stochastic behavior of HMMs and the simulated time series. Therefore, for reference, ${\bar{V}}_{{{\rm{R}}}}({{{\bf{H}}}}_{1},\ldots ,{{{\bf{H}}}}_{100})$ is computed, which gives the model-dependent stochastic variation of 2D_Pr_. In Figs. 6A–C and 7A–C, the score ${\bar{V}}_{{{\rm{D}}}}({{\bf{G}}},{{{\bf{H}}}}_{1},\ldots ,{{{\bf{H}}}}_{100})$ is in agreement with the deviation observed by visual inspection of the 2D_Diff_-histograms.

Furthermore, Hines and colleagues^54^ demonstrated the importance of estimating the scatter of the transition rates in addition to the goodness of the estimated model. They obtained the scatter by exploring the parameter space using Bayesian inference. It does not only provide confidence intervals for the rates, in addition, it indicates possible non-identifiability of transition rates associated with non-unique models if the scatter is not strictly confined^54^. Advancing this idea, a fit was introduced for Markov modeling of whole-cell patch-clamp data combined with fluorescence data^55^. This leads to the question of how to implement uncertainty quantification of the rates *k*_*ij*_ with our Deep Learning approach.

We found a solution that exploits the inherent capabilities of the algorithm, namely simulation and model prediction. The 100 2D-histograms simulated with the initial prediction used for computing ${\bar{V}}_{{{\rm{D}}}}({{\bf{G}}},{{{\bf{H}}}}_{1},\ldots ,{{{\bf{H}}}}_{100})$ and ${\bar{V}}_{{{\rm{R}}}}({{{\bf{H}}}}_{1},\ldots ,{{{\bf{H}}}}_{100})$ were used to make re-predictions, such that the parameter space surrounding the initial prediction is explored. This way, confidence intervals for the transition rates can be obtained (Figs. 6D–F and 7D–F). In addition, the Deep Learning approach can support the experimentalist by pointing out potential model non-identifiability if the confidence intervals for the transition rates are not confined. However, if the initial prediction is of poor quality, which can be estimated a priori using the volume deviation scores, the confidence intervals are of limited value (for an interpretation of the scores see the Supplementary Results and Supplementary Fig. 2). An alternative method to address this issue could be to compute ${\bar{V}}_{{{\rm{R}}}}({{{\bf{H}}}}_{1},\ldots ,{{{\bf{H}}}}_{100})$ using segmented experimental data, forming 2D-histograms for each segment to replace the 100 2D-histograms (${{{\bf{H}}}}_{1},\ldots ,{{{\bf{H}}}}_{100}$) of the predicted solutions. The drawback of this approach is that, unlike simulated data, the experimental time series has a limited duration.

Given the data-driven nature of our approach, an increase in the accuracy of the predicted models should be observed with more information available. This can be done in two ways. One is increasing the length of the underlying experimental time series, and the other is increasing the size of the training dataset, i.e. the number and length of simulated time series. When examining the former (Fig. 5F) we found that, as expected, the error score (RAE) was reduced insinuating that longer recordings will lead to more accurate predictions. The effect of increasing the amount of training data was examined in Fig. 3B, where the gain in accuracy is illustrated. Moreover, this elucidates the importance of using simulated time series as a basis for training the NNs, given that the overall simulated time of the dataset equates to years of recording time. This brings up the question if the computational constraints regarding simulation time (Table 2) could be alleviated. One possible approach to this is presented by ref. ^56^, where the authors use a generative adversarial network (GAN) to simulate a single-channel patch-clamp time series. Using a graphic accelerator, this algorithm is very efficient and could potentially generate massive amounts of data in a very short time. However, there are two reasons this method is not suitable for our approach. First, the GAN uses samples of real experimental time series as input. Hence, the ground truth of the outputted synthetic data is still unknown. Second, it is uncertain if the data simulated with the GAN is similar enough to real experimental data such that the NNs will not become confounded.

Thus, not only the amount of data is paramount but also its quality. To ensure that the simulated data is as similar as possible to real single-channel patch-clamp data, we derived an experimentally recorded step response, similarly to^30^. Additionally, we recorded noise using the patch-clamp setup, computed the power spectrum, and used the algorithm proposed by ref. ^31^ to generate a randomized noise series. At this point, the question arose on how to test the Deep Learning approach reliably without having access to sufficient amounts of labeled experimental data. The answer was by emulating the activity of an ion channel using the patch-clamp setup. To that end, we generated semi-synthetic single-channel patch-clamp data, similarly to ref. ^42^. To demonstrate the impact of the improvements (noise spectrum and step response) to the realism of the simulated data, we generated combinations of datasets where we substituted the experimentally derived step response and noise with their analytically computed counterparts and used them to train different NNs. The importance of the improvements to the simulation routine is visualized in Fig. 8, where it is apparent that the quality of the simulated data has achieved a sufficient similarity, to the experimentally recorded data. Furthermore, we speculate that the transformation of the time series into 2D-histograms further reduced any confounding details still present in the simulated data. Unfortunately, open-channel noise^22^ could not be included in the semi-synthetic time series generated with the patch-clamp setup due to technical reasons. If implemented in the simulation in a future version, we assume that the additional information contained in the time series could improve model prediction.

What are the requirements for setting up a time series analysis with this approach? To obtain optimal results, noise has to be recorded and an experimental step-response has to be acquired from the recording system. The derived noise spectrum and the step-response are then used by the simulation. A selection of Markov model topologies and the boundaries of the corresponding transition rates have to be defined, and a set of times series is simulated for each topology. Finally, the NNs are trained on the 2D-histograms derived from the simulated time series. Simulation and training are computationally demanding and are best carried out on an HPC cluster. However, the simulated data can be reused for training new NNs and the number of topologies could be expanded. The trained NNs can also be reused or shared with other groups if a similar recording system is utilized. While all these steps can be performed using the software provided online (see methods) and consulting this manuscript and the Supplementary Methods, a user-friendly frontend with a graphical user interface (GUI) would be desirable in the future.

For real-time prediction, the algorithm has to be integrated into the recording software. Idealization of a time series, generation of the 2D-histogram, and inference using the NNs (Table 2) are computationally lightweight and could be running in the background of a standard office computer. Computation of the error scores according to Figs. 6A–F and 7A–F can be computed at the end of a recording without significant delay.

In some experiments, different recordings need to be integrated into a single Markov model, e.g. using voltage steps to model potential dependence or using varying compound concentrations to model the dose dependence. The variation of the transition rates under these conditions could aid in reducing the unidentifiable space of the models. In these cases, a joint fit is often used and could be implemented in a future iteration of the proposed Deep Learning approach. How can this be implemented? Importantly, the algorithm is, in principle, capable of analyzing non-stationary data^17,20^. Two different approaches are proposed here. For each recorded time series with varying conditions, an individual model can be estimated initially, and transition rate dependencies can be derived post hoc by using a separate fit. The advantage is additional assumptions necessary for a joint fit, such as constraining all recordings to a single topology, do not interfere with the initial model estimations. The disadvantage could be a potential loss of modeling power. One possible way of implementing the equivalent of a joint fit would be to train an NN to receive a set of multiple 2D-histograms as input (e.g., one for each recording condition). A further constraint could be to couple specific transition rates of the HMMs used to simulate the time series such as to account for membrane potential or compound concentration. In this case, the disadvantage would be the additional effort to set up the model simulation and modify the architecture of the NNs.

In conclusion, due to its power regarding low SNR and fast gating, the ability to provide scores for the goodness of an obtained solution, and the ability to assist the experimenter with topology identification, we believe this to facilitate a rapid evaluation of single-channel patch-clamp recordings. Finally, the proposed Deep Learning approach could be extended to become applicable to data of other domains that follow Markovian behavior.

## Supplementary information

Supplementary Material

## Data Availability

All setfiles to generate the training data of Table 1 and the corresponding trained NNs used in the manuscript are available at Zenodo (10.5281/zenodo.12750594).

## Code availability

The source code in its current stage is available at Zenodo (10.5281/zenodo.12750594).
