# Supplementary material for: A deep learning approach to real-time Markov modeling of ion channel gating
Source: Commun Chem. 2024 Nov 30;7:280. doi: 10.1038/s42004-024-01369-y (PMC11608338; doi:10.1038/s42004-024-01369-y)
Supplement: Supplementary file 1 — Supplementary Material [file 42004_2024_1369_MOESM1_ESM.pdf]

A Deep Learning approach to real-time Markov modeling of ion channel gating

Efthymios Oikonomou[1], Yannick Juli[1], Rajkumar Reddy Kolan[1], Linda Kern[1], Thomas Gruber[2], Christian Alzheimer[1], Patrick Krauss[3], Andreas Maier[3], Tobias Huth[1]

[1]Institut für Physiologie und Pathophysiologie, Friedrich-Alexander-Universität Erlangen-Nürnberg, Erlangen, Germany.

[2]Erlangen National High Performance Computing Center, Friedrich-Alexander-Universität Erlangen-Nürnberg, Erlangen, Germany

[3]Pattern Recognition Lab, Friedrich-Alexander-Universität Erlangen-Nürnberg, Erlangen, Germany.

Correspondence

Tobias Huth, MD, PhD

Institut für Physiologie und Pathophysiologie

Friedrich-Alexander-Universität Erlangen-Nürnberg

Universitätsstr. 17

91054 Erlangen

Germany

tobias.huth@fau.de

+49 (0) 9131 8522495

Supplementary Results

Comparison with Quantify Unknown Biophysics (QUB) online

A comparison with QUB online[1–3], an analytical approach based on the idealized time series, showed that the predictions of the neural network are very robust related to time series with a comparable poor SNR and fast gating events. The spread (uncertainty) around the ground truth in the neural network's (NN) predictions is considerably smaller. It is important to note that in the deep learning approach, the valid prediction ranges for each transition rate are indirectly constrained by the training data (see Supplementary Fig. 1, legend). In contrast, QUB does not have such constraints, and its performance could potentially improve if they were to be implemented.

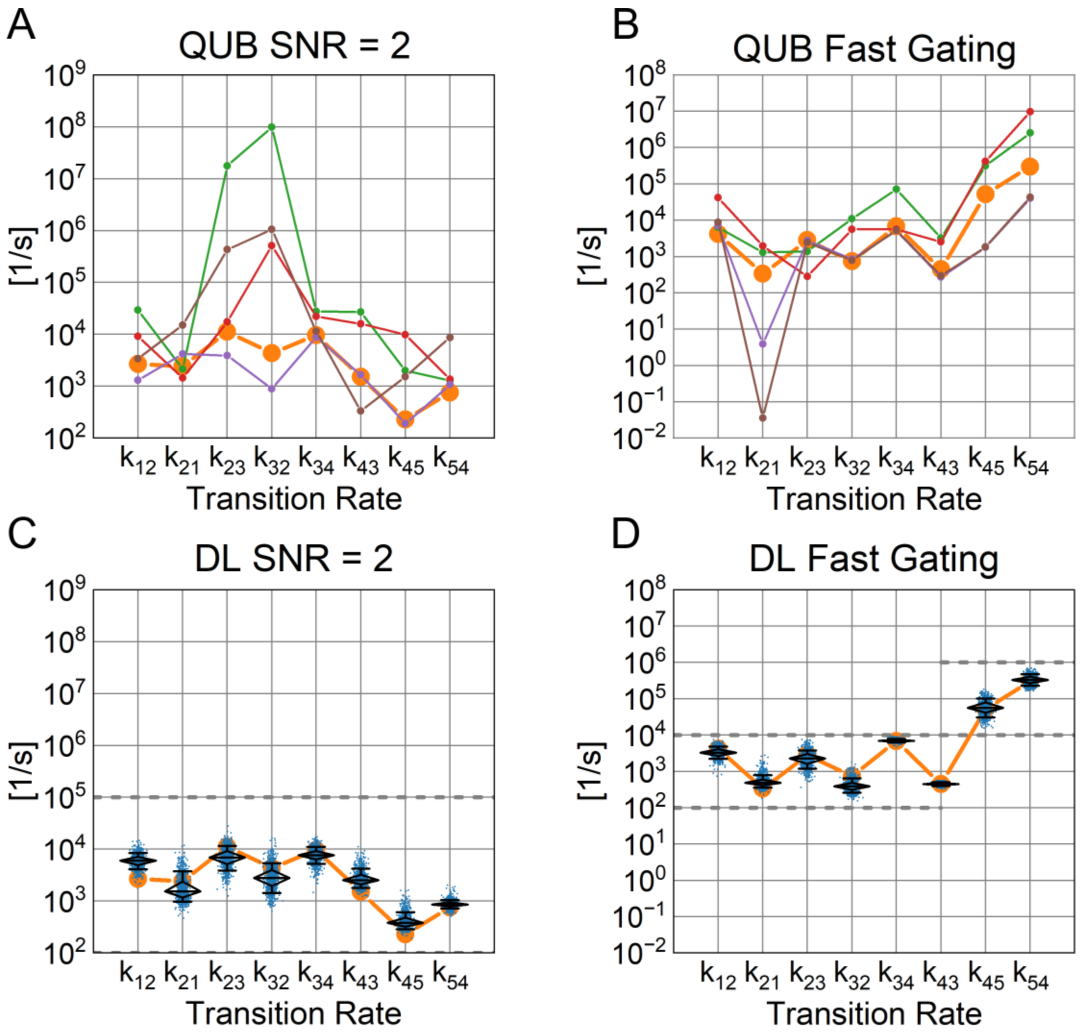

***Supplementary Figure 1*. Comparison of the Quantify Unknown Biophysics (QUB) online with the Deep Learning approach for (A,C) high noise and (B,D) fast-gating with a COCOC model.** QUB (https://qub.mandelics.com/online/) is based on the idealization of times series including a missed events correction and uses the Q-matrix. Time series with a length of **500,000 samples** (5 sec @ 100 kHz, low-pass filter cut off @ 10 kHz) were simulated using the 2D-Fit [4] with the predicted hidden Markov models (HMMs) from Fig. 6A of the manuscript (best prediction) for **(A,C)** and Fig. 7B (prediction below average) for **(B,D).** For each of the

two HMMs, 1,000 2D-histograms were simulated for the DL approach and four time series for QUB. Noise generation was done using the cell-model (patch) spectrum. The simulation, training, and analysis with the DL approach was done as described in the manuscript, using a Neural Network (NN) trained on 980,000 2D-histograms. In **(C)** and **(D)** the values of the predicted transition rates on the 1,000 2D-histograms using the DL approach are displayed with blue dots. The diamond indicates the median, 25, and 75 percentiles, while the whiskers denote the 10 and 90 percentiles. For QUB, the time series were idealized using its own provided detector [5], with a maximum number of re-estimations of 10, initialized with the correct COCOC topology and randomly drawn transition rates. The current and noise levels were fixed to the true value and were not re-estimated. For the estimation of the transition rates two search algorithms, were applied serially. First, the Simplex followed by a search routine that uses the Davidov-Fletcher-Powell (DFP) method. Each fit was commenced for 100 iterations and as stated in the options of the program using a max step of 1, a dx (num grad) of 10^{-7}, and a dead time of 0.01 (default). For each of the four time series, an ensemble of 10 fits was conducted, each time starting with re-randomized initial values for the transition rates. **The solutions with the highest likelihood for each time series (according to QUB) were plotted in (A) and (B)** (green, purple, red, and brown dots). Values connected with lines belong to the same predicted model. All random transition rates for the initialization were drawn from uniform logarithmic distributions sharing ranges with the HMMs used for simulating training datasets of the NNs. For **(A,C)** those are 100 s^{-1} to 100 ks^{-1} and for **(B,D)** the rates k_{12} to k_{43} are in the range 100 s^{-1} to 10 ks^{-1}, while k_{45} and k_{54} are between 10 ks^{-1} to 1 Ms^{-1} (grey dashed lines). The ground truth is indicated with the large orange dots connected with o lines.

Interpretation of the volume deviation scores and the scatter of the predicted rate constants

We examined three datasets in more detail, namely No. 2, No. 5, and No. 6 (Table 1), containing models of the linear COCOC topology with an SNR = 5 and an SNR = 2 for "regular" gating and fast gating, respectively (Supplementary Fig. 2). We computed $\bar{V}_{\mathrm{D}}(\mathbf{G}, \mathbf{H}_0, \mathbf{H}_1, \dots, \mathbf{H}_N)$, $\bar{V}_{\mathrm{D}}(\mathbf{G}, \mathbf{H}_0, \mathbf{H}_1, \dots, \mathbf{H}_N) - \bar{V}_{\mathrm{R}}(\mathbf{H}_0, \mathbf{H}_1, \dots, \mathbf{H}_N)$ and the uncertainty quantification of the transition rates for of the models included in the subset of the selected datasets (Supplementary Fig. 2). When gauging a prediction given by the NNs, all three scores are to be considered. A low value for $\bar{V}_{\mathrm{D}}(\mathbf{G}, \mathbf{H}_0, \mathbf{H}_1, \dots, \mathbf{H}_N)$, (orange line) indicates a good match of the 2D-histograms, which means that the underlying kinetic is captured successfully. The value of $\bar{V}_{\mathrm{D}}(\mathbf{G}, \mathbf{H}_0, \mathbf{H}_1, \dots, \mathbf{H}_N) - \bar{V}_{\mathrm{R}}(\mathbf{H}_0, \mathbf{H}_1, \dots, \mathbf{H}_N)$, which indicates the match of the observed and predicted kinetic when accounting for the stochastic variability of the predicted model should be low. As an orientation point we consider a RAE score (green line) below 0.6 as an acceptable. A small spread of the transition rates (top value of the blue area) would imply the uniqueness of the solution. Finally, one example where the RAE indicate a bad fitting result, albeit the scores indicating a good result, is given in Fig. 7C. The very rarely occurring bursts (red arrow in Fig. 7C and Supplementary Fig. 2) are statistically not represented in the experimental 2D-histogram ($2D_{GT}$). This results in fairly good values for $\bar{V}_{\mathrm{D}}(\mathbf{G}, \mathbf{H}_0, \mathbf{H}_1, \dots, \mathbf{H}_N)$ and $\bar{V}_{\mathrm{D}}(\mathbf{G}, \mathbf{H}_0, \mathbf{H}_1, \dots, \mathbf{H}_N) - \bar{V}_{\mathrm{R}}(\mathbf{H}_0, \mathbf{H}_1, \dots, \mathbf{H}_N)$, and a relatively low spread of the transition rates. Yet, the transition rates differ marked from the ground truth because of the missed burst events. In this cases comparison of the experimental time series and the simulated time series with the predicted model identifies the problem. In conclusion, positively stated, the algorithm offers the necessary tools to evaluate a predicted model.

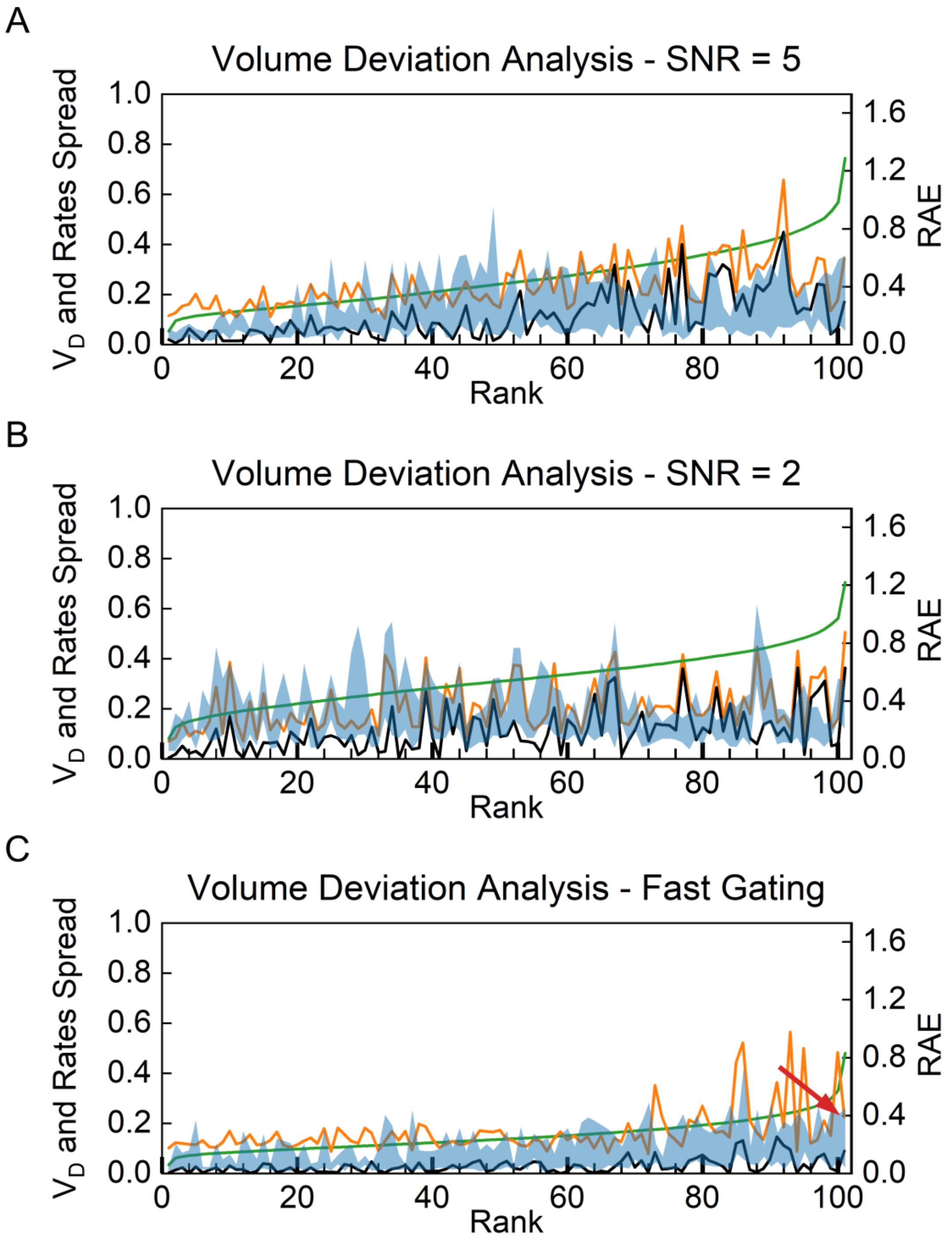

***Supplementary Figure 2*. Illustration of the volume deviation scores and uncertainty quantification of the re-predicted transition rates.** The volume deviation scores and the uncertainty of the predicted rates is plotted against the ranked RAE score for the test dataset splits of datasets No. 2, No. 5, and No. 6 (Table 1) in **(A)**,**(B)**,**(C)** respectively. Every 100th model out of the ranked test datasets was simulated 100 times and the analysis according to Fig. 6,7 was performed. The green line indicates the RAE of the predicted transition rates when compared to the ground truth rates. The orange graph shows the $\bar{V}_{\mathrm{D}}(\mathbf{G}, \mathbf{H}_0, \mathbf{H}_1, \dots, \mathbf{H}_N)$ score (eq. 6) and the black graph the difference $\bar{V}_{\mathrm{D}}(\mathbf{G}, \mathbf{H}_0, \mathbf{H}_1, \dots, \mathbf{H}_N) - \bar{V}_{\mathrm{R}}(\mathbf{H}_0, \mathbf{H}_1, \dots, \mathbf{H}_N)$. Additionally, the standard deviations of the logarithmically scaled predicted transition rates were computed for each of the eight transition rates of the 101 models. The blue area marks the span between the smallest and largest of those standard deviations for each model. For the red arrow, see text.

Supplementary Methods

Reproducing the data and setting up the experiments

The following section is intended to give instructions on how to reproduce the results of this manuscript. It also serves as a template to start your own analysis (supported by the README.txt file in the same folder). The software is published at Zenodo (10.5281/zenodo.12750594). For additional information and support please contact the authors directly.

<u>Important note</u>: **The instructions for "*Generation of the training datasets*" and "*Neural network training*" are meant to be used with appropriate HPC resources (for hardware specifications see methods) and will either take considerable time or might not run at all on a desktop PC / workstation.**

Software and compilation

- Linux OS

- Open MPI 4.1.2 with compiler gcc11.2.0

- Python 3.9

- tensorflow 2.7

- The `2DFit64` can be compiled using the provided compile.sh file found in the "`2DFit`" folder.

Generation of the training datasets

For the simulation of the training datasets an `.ini` and a `.set` file containing the information about the model, noise, and step response are required. The `.ini` and `.set` files for the generation of all datasets listed in Table 1 are provided in the folder "`2DFit/preset_set_and_ini_files`" and are enumerated as in Table 1. To simulate a dataset follow the next steps:

1. Move the `.ini` file to the same folder as the executable (`2DFit64`) and define the path to the `.set` file within the `.ini` file (line 95).
2. Execute `2DFit64` and after the simulation is complete, find the `.dat` (binary) file in the same folder as the `.set` file.
3. Open the "`Save_as_numpy.py`" script and set the *path to the simulated binary file, the target path to the folder were the resulting NumPy file should be saved,* and the *number of*

histograms to save from the simulated binary file (out of 1,000,000 which are simulated by default).

4. Execute the "`Save_as_numpy.py`" script to transform the `.dat` file into a NumPy file. The resulting NumPy file contains the 2D-histograms with the corresponding transition rates and is used to train the neural network (NN).

Neural network training

The 2DDL repository contains all required scripts to train and save the NNs. For training a transition rate NN follow the next steps:

1. Open the "`Deep_dwell_time_regression.py`" file ("`2DDL`" folder) and set the *path to the training dataset* and the *name of the training dataset* simulated in the previous section. Additionally, set the *name of the result folder* and the *path to the result folder,* were the trained NN is to be saved. In case of a symmetric topology (see methods) set the "`rearrange_labels`" variable to `True.`
2. Execute the "`Deep_dwell_time_regression.py`" script.

To train a topology estimation NN, follow the next steps:

1. Simulate a number of different datasets, each including a different topology using the pipeline described above.
2. List the names of the resulting NumPy datasets in the "`topology_datasets.txt`" file, as exemplified in the already existing file.
3. Open the "`Deep_dwell_time_topology.py`" file and set the *path to the training datasets (containing all the different Markov topologies).* Additionally, set the *name of the result folder* and the *path to the result folder,* were the trained NN is to be saved.
4. Execute the "`Deep_dwell_time_topology.py`" script.*Generation of 2D-histograms*

Given a time series in `.txt (ASCII)` format to be analyzed, the 2D-histogram of the time series can be computed by following the next steps:

1. Open the "`2D_histogram_generator.py`" script ("`2DDL`" folder) and set the *path to the time series and save location for 2D-histogram.* Also, set the *name of the file of the time series* and the *name of the resulting NumPy file of the 2D-histogram.*
2. Set the *higher amplitude level* and the *lower amplitude level* as well as the *standard deviation of the noise* (used to generate the idealized time series)
3. Execute the "`2D_histogram_generator.py`" script.

Model inference

Using a trained topology estimation NN, the topology can be inferred with the computed 2D-histogram, by following the steps:

1. Open the "`Inference_topology.py`" script ("`2DDL`" folder) and set the *path to the trained NN,* as well as the *path to the 2D-histogram* and the *name of the 2D-histogram.*
2. Execute the "`Inference_topology.py`" script.

Next, using the trained transition rates estimation NN, the transition rates can be inferred with the computed 2D-histogram, by following the steps:

1. Open the "`Inference_regression.py`" script ("`2DDL`" folder) and set the *path to the trained NN,* as well as the *path to the 2D-histogram* and the *name of the 2D-histogram.*
2. Execute the "`Inference_regression.py`" script.

Furthermore, all trained NNs used in the manuscript can be found in the folder "`trained_NNs`", enumerated by the datasets they were trained on (Table 1).

Simulation of a time series from a specific model, generation of the corresponding 2D-histogram and estimation of the prediction error

For the simulation of a time series given a specific model and computation of its 2D-histogram, follow these steps:

1. Move the `.ini` file ("`2DFit/fixed_transition_rates/single_time_series`" folder) to the same folder as the executable (`2DFit64`) and define the path to the `.set` file within the `.ini` file (line 95). The provided `.set` file is an example for the ground truth model of Fig. 6A. For further details consult the README file.
2. Execute `2DFit64`.
3. To convert the resulting `.dat` (binary) file to a `.txt` file, open the "`Time_series_to_txt.py`" script ("`2DFit`" folder) and define the *path to the binary file* and the *path to the save location of the text file* and then execute it.
4. To compute the 2D-histogram of the simulated time series (as stated above), open the "`2D_histogram_generator.py`" script ("`2DDL`" folder) and set the *path to the time series and save location for 2D-histogram.* Also, set the *name of the file of the time series* and the *name of the resulting NumPy file of the 2D-histogram* and execute the script.

Error calculation: To simulate 100 2D-histograms given a specific model, follow these steps:

1. Go to the "2DFit/fixed_transition_rates/100_histograms" folder. The provided .set file is an example for the predicted model of Fig. 6A. For further details consult the README file.
2. Move the .ini file to the same folder as the executable (2DFit64) and define the path to the .set file within the .ini file (line 95).
3. Execute 2DFit64 and after the simulation is complete, find the .dat (binary) file in the same folder as the .set file.
4. Open the "Save_as_numpy.py" script ("2DFit" folder) and set the *path to the simulated binary file, the target path to the folder were the resulting NumPy file should be saved,* and the *number of histograms to save from the simulated binary file* to 100.
5. Execute the "Save_as_numpy.py" script to transform the .dat file into a NumPy file. The resulting NumPy file contains the 2D-histograms with the corresponding transition rates

To re-estimate the model using the 100 simulated 2D-histograms, follow these steps:

1. Open the "Inference_regression_reestimation.py" script ("2DDL" folder) and set the *path to the trained NN,* as well as the *path to the 2D-histogram* and the *name of the 2D-histogram.*
2. Execute the "Inference_regression_reestimation.py" script.

Computation of the volume deviation scores

The volume deviation and volume reference scores (eq. 5,6) can be computed using the python scripts, respectively:

- "Volume_deviation.py" ("2DDL" folder) set the *path to the 2D-histograms,* the *name of the predicted 2D-histograms file, and the name of the ground truth 2D-histogram file*. Execute the script.
- "Volume_ref_computation.py" ("2DDL" folder) set the *path to the 2D-histograms* and the *name of the predicted 2D-histograms file (e.g. containing 100* simulated 2D-histograms). Execute the script.

Semi-synthetic data repository and example 2D-histogram

The semi-synthetic datasets used in Fig. 9 of the manuscript are provided in folder "2DDL/semi_synthetic_data". Additionally, an example 2D-histogram of the linear five-state

COCOC topology with rates (16000, 8000, 4000, 2000, 1000, 500, 250, 125) s^{-1} simulated using an SNR=5, the experimental step response, and spectral patch noise (recorded with an Axopatch 200B) is provided in NumPy format in the “`2DDL/2D_histograms`” folder.

Supplementary References

1. Hawkes, A. G., Jalali, A. & Colquhoun, D. Asymptotic distributions of apparent open times and shut times in a single channel record allowing for the omission of brief events. *Philos. Trans. R. Soc. Lond. B. Biol. Sci.* **337**, 383–404 (1992).

2. Qin, F., Auerbach, A. & Sachs, F. Estimating single-channel kinetic parameters from idealized patch-clamp data containing missed events. *Biophys. J.* **70**, 264–80 (1996).

3. Qin, F., Auerbach, A. & Sachs, F. Maximum likelihood estimation of aggregated Markov processes. *Proceedings. Biol. Sci.* **264**, 375–83 (1997).

4. Oikonomou, E. *et al.* 2D-dwell-time analysis with simulations of ion-channel gating using high-performance computing. *Biophys. J.* **122**, 1287–1300 (2023).

5. Qin, F. Restoration of Single-Channel Currents Using the Segmental k-Means Method Based on Hidden Markov Modeling. *Biophys. J.* **86**, 1488–1501 (2004).
